# Longitudinal trajectories of hematological indices and serum metalloproteinases-2 and 9 over 1 year after moderate and severe COVID-19

**DOI:** 10.3389/fmed.2026.1824883

**Published:** 2026-06-12

**Authors:** Rebecca Salomão, Gabriel Pasquarelli-do-Nascimento, Mayara Ananias, Victoria Assis, Leandro Gomes de Jesus Ferreira, Isabella da Silva Almeida, Leandra Silva, Katiane Tostes, Bruna Pereira Sorroche, Lidia Maria Rebolho Batista Arantes, Kelly Grace Magalhães, Liliana Torcoroma García, Rochelle Rocha Costa, João Luiz Quagliotti Durigan, Rita de Cássia Marqueti

**Affiliations:** 1 Laboratory of Molecular Analysis, Graduate Program Health Sciences and Technologies, Faculty of Health Sciences and Technologies, University of Brasilia, Brasilia, Brazil; 2 Laboratory of Muscle and Tendon Plasticity, Graduate Program in Rehabilitation Science, Faculty of Health Sciences and Technologies, University of Brasilia, Brasilia, Brazil; 3 Faculty of Health Sciences and Technologies, University of Brasilia, Brasilia, Brazil; 4 Molecular Oncology Research Center, Barretos Cancer Hospital, Barretos, Brazil; 5 Laboratory of Immunology and Inflammation, Department of Cellular Biology, University of Brasília, Brasília, Brazil; 6 Infectious Disease Postgraduate Program, Universidad de Santander, Bucaramanga, Colombia; 7 Department of Physical Education, Graduate Program in Physical Education, University of Brasilia, Brasilia, Brazil

**Keywords:** COVID-19, hematological indices, inflammatory mediators, longitudinal study, metalloproteinases

## Abstract

**Introduction:**

The long-term clinical burden of Coronavirus disease (COVID-19) remains substantial, yet one-year cohort evidence linking blood-based biomarkers to persistent sequelae is limited.

**Methods:**

We conducted a longitudinal study in adults aged 18–80 years who were classified as moderate or severe COVID-19 or non-COVID-19 controls. Blood was collected at four time points through 360 days after infection or hospital discharge. Cytokines were quantified by flow cytometry. MMP-2 and MMP-9 levels and activity were assessed by gelatin zymography. Hematological parameters were measured in an accredited clinical laboratory. Longitudinal effects were evaluated using generalized estimating equations.

**Results:**

Compared with moderate cases and controls, participants who experienced severe acute disease showed persistent immune dysregulation and systemic inflammation at approximately 1 year, with higher concentrations of inflammatory mediators and cytokines and sustained elevations in MMP-2 and MMP-9. Complete blood count–derived indices were also altered over time in severe cases, including the aggregate index of systemic inflammation, the C-reactive protein to lymphocyte ratio, the neutrophil to platelet ratio, and the systemic inflammation response index, together with red cell distribution width, while renal function tests, hepatic enzymes, and muscle injury markers were largely stable across groups.

**Discussion:**

These findings delineate a persistent inflammatory and matrix-remodeling signature up to 1 year after severe COVID-19, based on longitudinal biomarker profiles rather than symptom-defined long COVID-19 outcomes. This biomarker panel may help to inform future studies of post-acute risk stratification and targeted interventions, but prospective prognostic validation and clinical endpoint data are still required.

**Clinical trial registration:**

https://clinicaltrials.gov/study/NCT04961255?term=NCT04961255&rank=1, NCT04961255.

## Introduction

The Coronavirus Disease (COVID-19) pandemic has had a profound and lasting impact on the world, resulting in approximately 7 million recorded deaths to date and leaving deep social, economic, and long-term health consequences (1). The acute Severe Acute Respiratory Syndrome Coronavirus 2 (SARS-CoV-2) infection activates the innate immune system, triggering antiviral responses (2). In severe cases, an excessive inflammatory reaction, or “cytokine storm,” causes secondary damage to tissues and contributes to acute respiratory distress syndrome (ARDS), pulmonary failure, and multi-organ dysfunction (3).

Blood biomarkers and hemogram-derived ratios provide key insights into inflammation, immune dysregulation, and tissue recovery in acute COVID-19, and can be explored to assess disease severity, and monitor recovery (3–22). Acute COVID-19 has been associated with a range of organ-specific complications, including renal function decline (23), muscle injury (24), cardiovascular involvement (25), and immune imbalances and inflammation (3–22). In addition, matrix metalloproteinases (MMPs), involved in extracellular matrix (ECM) remodeling, have emerged as important mediators in COVID-19 pathogenesis (26–28). In this study, “long-term COVID-19 manifestations” are operationally defined as persistent alterations in inflammatory, hematological, and matrix remodeling biomarkers observed during the post-acute phase (≥21 days after symptom onset or hospital discharge), rather than clinically adjudicated symptom-based outcomes. Our focus was therefore on biological trajectories following acute infection.

Elevated levels of MMPs have been observed in acute COVID-19 cases, where they contribute to tissue dysfunction and may drive persistent symptoms through the promotion of chronic inflammation and ECM degradation (29, 30). Other studies have also reported increased MMP gene expression in cerebrospinal fluid (CSF) (31), myocardium (32), and lungs (33), indicating their potential involvement in long-term complications beyond the acute phase of the disease.

Authors suggest the existence of a large percentage of patients who recovered from the acute phase of SARS-CoV-2 infection but continue to experience persistent symptoms or develop new health issues without an alternative diagnosis over time (34). COVID-19 long-term consequences are associated with decreased quality of life and rising unemployment rates. Affected individuals exhibit a wide range of disabling and debilitating symptoms, which impose a relevant economic burden to global population because of the alarming number of affected individuals unable to return to work (34–36). This condition poses medical, epidemiological, and sociopolitical challenges, affecting patients’ quality of life and straining public health systems and policies worldwide.

Various authors investigating individuals experiencing long-term manifestations associated with severe acute COVID-19 have detected disrupted hematological parameters, including several inflammatory biomarkers and MMPs. Studies have also linked these parameters to the risk of the occurrence of persistent hematological alterations (5, 6, 28, 37). However, there is limited information regarding these markers in subjects who were affected by moderate and severe acute-COVID-19 1 year after infection. Long-term manifestations associated with severe acute COVID-19 remain a global health crisis, characterized by multi-system chronic conditions such as fatigue, cognitive impairment, cardiovascular abnormalities, and renal dysfunction. Understanding the mechanisms driving these sequelae, particularly the roles of immune dysregulation, persistent inflammation, and tissue damage, is critical for identifying therapeutic targets to mitigate chronic disease progression. However, longitudinal studies tracking blood biomarkers over extended periods (e.g., 1 year) in moderate and severe COVID patients are scarce, limiting prognostic and diagnostic advancements. To address this gap, our study investigated blood biomarker profiles over a one-year follow-up in a cohort of moderate and severe COVID-19 patients. We focused on markers of renal function, muscle injury, cardiovascular risk, immune derangement, chronic inflammation, and matrix remodeling to characterize longitudinal associations between these biomarker profiles and post-acute phases of COVID-19. We hypothesized that individuals who had experienced severe acute COVID-19 would display more pronounced and sustained dysregulation of these biomarkers over time compared with individuals with moderate disease and non-COVID controls.

## Materials and methods

### Study design

We conducted a longitudinal observational study comparing participants with moderate or severe COVID-19 to a control group. The study was approved by the university Research Ethics Committee (CAAE 45043821.0.0000.8093), and all participants or their family members provided informed consent after receiving details about the study. The trial is registered at ClinicalTrials.gov (NCT04961255). This study adhered to the ethical principles for human experimentation as outlined in the World Medical Association’s Declaration of Helsinki.

### Participants

Participants of both sexes, aged between 18 and 80 years, who tested positive for COVID-19 were recruited. The considerations of Gandhi and colleagues were used for patient stratification (38). This study included three groups: (a) Moderate COVID-19 group: individuals who tested positive for COVID-19 exhibited symptoms such as dry cough, rhinorrhea, sore throat, diffuse body pain, and persistent hyperthermia, without signs of hypoxemia and without requiring hospital admission. (b) Severe COVID-19 group: individuals who also tested positive for COVID-19 and presented one or more of the symptoms described above, along with hypoxemia [oxygen saturation – (SPO_2_) ≤ 93%] requiring hospitalization with or without intubation. (c) Control group: individuals who either tested negative or remained asymptomatic during the pandemic (39). These participants were selected to match the COVID-19 groups in terms of anthropometric and demographic characteristics. COVID-19 diagnosis was confirmed using RT-PCR molecular testing, or immunochromatographic assays for antibody and antigen detection.

Exclusion criteria included pregnancy, presence of pain or edema, history of cancer, skin lesions, ankylosing spondylitis, rheumatoid arthritis, severe cardiovascular disease, or advanced chronic obstructive pulmonary disease (COPD).

### Demographic and clinical data

This study did not include formal clinical assessment of long COVID-19 symptoms (e.g., fatigue, dyspnea, cognitive impairment). Instead, participants were followed longitudinally to evaluate biomarker dynamics after acute COVID-19, stratified by initial disease severity. Demographic and clinical data, including sex, age, body mass index, and comorbidities (hypertension, diabetes, dyslipidemia, mental health disorders, and asthma), were collected through a structured questionnaire specifically developed by the research team. Participants in the severe COVID-19 group also provided detailed information regarding their infection history. Additional variables assessed included hospitalization duration, period of intensive care unit (ICU) hospitalization, vaccination status, smoking history, and physical activity levels. These baseline assessments were essential to contextualize the study findings and to identify factors that may influence long-term inflammation, immune responses, and recovery trajectories.

### Study workflow

**Figure 1:**
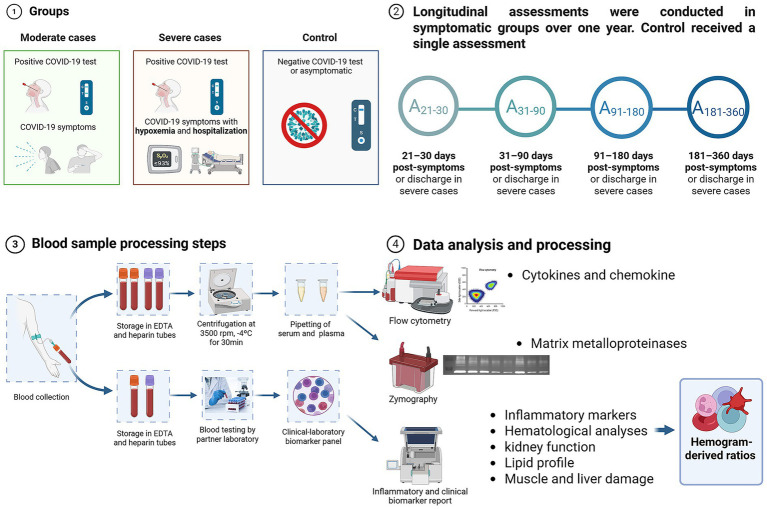
Experimental design. Schematic representation of the experimental design of this study, depicting the experimental groups, frequency of assessments, sample collection and processing, and data analysis. **(1)** This study included individuals with moderate COVID-19 symptoms, individuals with severe symptoms, and a control group composed of uninfected or asymptomatic participants. **(2)** We conducted longitudinal assessments by collecting blood samples at four time points for post-COVID-19 participants, corresponding to 21–30, 31–90, 91–180, and 181–360 days after symptom onset or hospital discharge for severe cases. Control participants were assessed at a single time point. **(3)** Peripheral venous blood was collected, centrifuged, and aliquots of serum and plasma were prepared for multiple analyses. **(4)** Cytokines and chemokine were quantified by flow cytometry, and matrix metalloproteinases were analyzed using zymography. Other inflammatory markers and biomarkers of kidney function, liver damage, cardiovascular risk, and immune dysregulation were investigated using the ELISA method. Created in BioRender. Salomão (2026) https://BioRender.com/yxs9b5f. License (Agreement number): TS29SIQ8RW.

Following recruitment, four assessments were conducted over 1 year: A_21–30:_ between the 21st and 30th days after the onset of symptoms or hospital discharge, A_31–90:_ between 31 and 90 days after the onset of symptoms or hospital discharge, A_91–180:_ between 91 and 180 days after the onset of symptoms or hospital discharge, A_181–360_: between 181 and 360 days after the onset of symptoms or hospital discharge (Figure 1). The time origin was defined as the earliest available reference between symptom onset (for non-hospitalized participants) and hospital discharge (for hospitalized participants), reflecting the transition into the post-acute phase. This approach was adopted to capture recovery trajectories, although it introduces heterogeneity in time-zero definitions. The control group was assessed only once for comparative purposes, as several participants contracted COVID-19 after the initial evaluation, making it unfeasible to retain them as longitudinal controls during the outbreak.

### Blood collection

Peripheral venous blood was collected from each participant by a research-affiliated nurse between 7:00 a.m. and 8:00 a.m. Two 8 mL serum separator tubes and two 8 mL EDTA tubes were used, along with one heparinized and one additional EDTA tube separate for clinical analyses. Serum and plasma were separated by centrifugation (Hermle Z 366 K, Germany), aliquoted (2 mL each), and stored at −80 °C for later analysis.

### Quantification of cytokines by flow cytometry technique

Cytokine levels were analyzed by Cytometric Bead Array (CBA) technology. The BD Human Inflammatory Cytokines Kit was used to measure Interleukin (IL)-8, IL-1β, IL-6, IL-10, IL-12p70, and Tumor Necrosis Factor (TNF-*α*), while the BD Human Flex Set assay was employed to quantify Interferon-gamma (IFN-*γ*) and IL-17A. For each analysis, 50 μL of plasma was used.

Plasma samples were incubated with a mixture of capture beads, each coated with a specific antibody against a target cytokine and distinguished by unique fluorescence intensities, to allow protein binding. After incubation, a washing step was performed to remove unbound proteins and minimize background interference. Phycoerythrin (PE)-conjugated detection antibodies were added to the captured cytokines, forming a sandwich complex that enhances the fluorescent signal. Samples were analyzed using a BD Accuri C6 Flow Cytometer (Figure 1), with bead populations identified based on light scatter properties and fluorescence intensity to quantify cytokine levels. We acquired a total of 300 events per cytokine. Using the Cytometric Bead Array software, fluorescence intensity values were converted into cytokine concentrations in the samples by referencing a standard curve with known concentrations. Results were exported for subsequent statistical analyses.

### Analysis of laboratory biomarkers

The analysis of blood elements and the quantification of biomarkers of inflammation, kidney function, liver and muscle damage, and lipid profile were conducted by a collaborating diagnostic laboratory (Sabin Laboratory^®^), as illustrated in Figure 1.

### Hematological indices

Hematological indices were derived from complete blood count (CBC) and biochemical test results obtained from an accredited diagnostic laboratory (Figure 1). Calculations followed established methods described in the literature (40–43). These indices were used to assess systemic inflammation and immune status and were computed using standardized formulas in electronic spreadsheets. A summary of each index and its corresponding formula is provided in Table 1.

**Table 1:** Hematological indices and their respective formulas.

Index	Formula
Aggregate index of systemic inflammation (AISI)	(Neutrophils × monocytes × platelets)/lymphocytes
C-reactive protein to lymphocyte ratio (CLR)	C-reactive protein/lymphocytes
Lymphocyte to C-reactive protein ratio (LCR)	Lymphocytes/C-reactive protein
Lymphocyte to monocyte ratio (LMR)	Lymphocytes/monocytes
Monocyte to lymphocyte ratio (MLR)	Monocytes/lymphocytes
Monocyte to Neutrophil Ratio (MNR)	Monocytes/neutrophils
Neutrophil to lymphocyte and platelet ratio (NLPR)	Neutrophils/(lymphocytes + platelets)
Neutrophil to lymphocyte ratio (NLR)	Neutrophils/lymphocytes
Neutrophil to platelet ratio (NPR)	Neutrophils/platelets
Platelet to lymphocyte ratio (PLR)	Platelets/lymphocytes
Reticulocyte to platelet ratio (RPR)	Reticulocytes/platelets
Systemic inflammation index (SII)	(Neutrophils × platelets)/lymphocytes
Systemic inflammatory response index (SIRI)	(Neutrophils × monocytes)/lymphocytes

The table summarizes the formulas used to calculate various hematological indices based on complete blood count (CBC) and biochemical parameters.

### Quantification of MMP levels by Zymography technique

To measure metalloprotease activity in the blood, Zymography technique was performed based on the procedure described previously (44). Total of 0.5 μL of 8% Sodium Dodecyl Sulfate (SDS) was combined with 0.5 μL of plasma. After vortexing, 9 μL of sample buffer was added without *β*-mercaptoethanol (reducing agent) containing SDS (20%). Samples were resolved by electrophoresis on a 7.5% polyacrylamide gel containing 10% sodium dodecyl sulfate (SDS) and gelatin at a final concentration of 1 mg/mL, following the Abcam protocol (45). To identify latent, intermediate, and active MMP isoforms, 5 μL of the pre-stained molecular mass marker (Thermo Scientific™ PageRuler™ Prestained Protein Ladder, Thermo Scientific, Inc., Lithuania) was added to the gel. After electrophoresis, we washed the gel twice.

for 20 min in a 2.5% Triton X-100 solution to remove SDS. Then, we incubated the gel in substrate buffer (50 mM Tris–HCl, pH 8.0, 2.5 mM CaCl₂, 0.02% NaN₃, 10 mM ZnCl₂) at 37 °C for 18 h to allow enzymatic activity. Next, we stained the gel with Coomassie Brilliant Blue R-250 (Bio-Rad) for 120 min, and destained with 10% methanol and 10% acetic acid. Densitometric analysis was performed using the InGenius imaging system with GeneSys for image acquisition and GeneTools software for band quantification (Syngene, Frederick, MD, United States), as shown in Figure 1.

### Statistical analyses

To verify the differences between groups and assessments, we adopted the Generalized Estimating Equations (GEE) method, using “group” and “assessment” as factors. The analyses were designed to assess longitudinal associations and temporal patterns using GEE models; no predictive modeling, risk stratification, or validation procedures were performed. We evaluated the interaction between “group” and “assessment” to detect differences across assessments for time-dependent variables, such as age, weight, and body mass index (BMI). The independence model criterion (QIC), as proposed by Pan (46), was used to determine the optimal functional correlation structure for the GEE analyses. We used this criterion to select the optimal working correlation structure in GEE analyses. The least significant difference (LSD) method served as a *post hoc* test to identify specific differences. For data analysis related to sample characterization, we also applied the GEE method, considering the factor “group” to compare the means of continuous variables across the three groups. We analyzed categorical variables using the Chi-Square Test. We set statistical significance at *α* < 0.05 and performed the analyses using SPSS version 22.0 (IBM Corporation, Armonk, NY, United States). We created graphical representations using RStudio (RStudio, PBC, Boston, MA, United States).

GEE were selected instead of repeated-measures ANOVA because the present study involved correlated longitudinal observations within participants and a partially unbalanced design, with repeated follow-up in the COVID-19 groups and a single assessment in the control group. GEE was considered more appropriate for estimating population-averaged effects of group, assessment, and group-by-assessment interaction under these conditions.

A formal *a priori* sample size calculation was not feasible because, at the time of study design, there were no robust longitudinal data on the investigated biomarkers in post-acute COVID-19 to support reliable assumptions regarding effect size, variability, correlation structure, and attrition across repeated assessments.

## Results

To identify factors associated with post-acute biomarker alterations after COVID-19, demographic and health-related characteristics were assessed (Table 2). Participants who had experienced severe acute COVID-19 were older and had higher body weight and BMI compared with the control and moderate groups. Severe cases also showed a higher prevalence of comorbidities, including hypertension, diabetes, and dyslipidemia. Moreover, physical activity levels were significantly lower in the severe group across all time points.

**Table 2:** Characterization of patients grouped according to disease severity (*n* = 145).

Characteristics	Groups			*p*-value
	Control	Moderate COVID	Severe COVID	
	(*n* = 30)	(*n* = 22)	(*n* = 18)	
	Mean (CI 95%)	Mean (CI 95%)	Mean (CI 95%)	
Sex (n)				0.988
Male	13	10	8	
Female	17	12	10	
Age (years)				
A_21–30_	46.53 (42.10–51.43)	38.27 (33.96–43.13)^*^	50.83 (45.19–57.18)^#^	0.001
A_31–90_	46.53 (42.10–51.43)	38.36 (34.04–43.23)^*^	51.11 (45.52–57.39)^#^	
A_91–180_	46.53 (42.10–51.43)	38.68 (34.33–43.59)^*^	51.28 (45.60–57.66)^#^	
A_181–360_	46.53 (42.10–51.43)	39.18 (34.90–43.99)^*^	51.89 (46.22–58.25)^#^	
Body mass (kg)				
A_21–30_	69.54 (65.07–74.31)	71.28 (64.14–79.21)	83.08 (75.91–90.92)^# *^	0.001
A_31–90_	69.54 (65.07–74.31)	71.57 (64.50–79.42)	84.89 (77.27–93.25)^a # *^	
A_91–180_	69.54 (65.07–74.31)	72.32 (65.14–80.28)	85.43 (77.51–94.15)^a # *^	
A_181–360_	69.54 (65.07–74.31)	73.12 (65.96–81.08) ^a, c^	87.49 (79.89–95.81)^a, b, c # *^	
Stature (m)	1.66 (1.63–1.69)	1.66 (1.62–1.70)	1.61 (1.55–1.66)	0.206
BMI (kg/m^2^)				
A_21–30_	24.91 (23.85–26.02)	25.55 (23.51–27.77)	31.94 (29.70–34.35)^# *^	0.001
A_31–90_	24.91 (23.85–26.02)	25.53 (23.46–27.77)	32.55 (30.34–34.91)^# *^	
A_91–180_	24.91 (23.85–26.02)	25.89 (23.76–28.20)	32.72 (30.45–35.16)^# *^	
A_181–360_	24.91 (23.85–26.02)	26.13 (24.10–28.33)^a, b^	33.62 (31.55–35.83)^a, b, c # *^	
Comorbidities (n)				
Hypertension	5	1	12 ^#^	0.001
Diabetes	0	1	7 *	0.001
Dyslipidemia	6	3	9 ^#^	0.021
Depression	0	1	5*	0.003
Anxiety	1	5	3	0.102
Panic Syndrome	0	1	2	0.183
Asthma	0	4	2	0.062
Hospital stay period (days)	N. A	N. A	38.38 (27.15–49.62)	N. A
ICU stay period (days)	N. A	N. A	21.44 (13.72–29.16)	N. A
COVID vaccine before infection (n) for moderate and severe groups and COVID vaccine at the time of evaluation for control group (n)				
Yes	28	22	16	0.298
No	2	0	2	
Number of doses	3.50 (3.25–3.77)	2.14 (1.89–2.41)^*^	2.00 (1.72–2.32)^*^	0.001
Current smoking (n)				
Yes	1	1	0	0.677
No	29	21	18	
Time (Years)	0.03 (0.00–0.06)	0.02 (0.00–0.05)	0.00 (0.00–0.00)	
Previous smoking (n)				
Yes	5	7	6	0.323
No	25	15	12	
Time (years)	16.60 (8.40–32.79)	15.85 (11.00–22.85)	27.16 (19.67–37.51)	0.076
Interruption time (years)	24.40 (19.34–30.78)	10.37 (5.45–19.72)^*^	11.63 (5.77–23.42)^*^	0.011
Engagement in physical activity before infection (n) and at the time of the assessment for the control group				
Yes	19	9	5^*^	0.045
No	11	13	13	
Engagement in physical activity at the time of assessment				
A_21–30_				0.001
Yes	19	8	3^#*^	
No	11	14	15	
A_31–90_				
Yes	19	14	5^#*^	
No	11	8	13	
A_91–180_				
Yes	19	17	5^#*^	
No	11	5	13	
A_181–360_				
Yes	19	14	4^#*^	
No	11	8	14	
Post-infection physiotherapy (n)				
Yes	N. A	0	13^#^	0.001
No	N. A	22	5	

NA, not applicable. A_21–30_, assessment carried out between 21 and 30 days after the onset of symptoms or hospital discharge for severe COVID, A_31–90_, assessment carried out between 31 and 90 days after the onset of symptoms or hospital discharge for severe COVID, A_91–180_, assessment carried out between 91 and 180 days after the onset of symptoms or hospital discharge for severe COVID, and A_181–360_, between 181 and 360 days after the onset of symptoms or hospital discharge for severe COVID. Control group was evaluated only once. * Different from the control group in the respective assessment (*p* < 0.05); # Different from the moderate COVID in the respective assessment (*p* < 0.05); a Different from A_21–30_ (*p* < 0.05) within group; b Different from A_31–90_ (*p* < 0.05) within group; c Different from A_91–180_ (*p* < 0.05) within group.

### Cytokines levels

Considering that COVID-19 is an inflammatory disease, we analyzed cytokine levels to better understand the immune response associated with persistent symptoms up to one-year post-infection (Table 3). IL-6 and IL-8 levels were significantly elevated in the severe group compared to the moderate and control groups, although no changes were observed over time. TNF-*α* showed a significant interaction between group and timepoint, with decreased levels in the severe group compared to control and moderate at the later stage (A_91–180_). IL-1β concentrations were higher in the severe and moderate groups relative to the control group, while IFN-*γ* was elevated in the severe group at the early timepoint (A_21–30_). No significant differences were found for IL-10 or IL-12p70 across groups or assessments.

**Table 3:** Cytokine, chemokine and inflammatory markers levels in different disease severity and time points of sample collection.

Cytokines		A_21–30_	A_31–90_	A_91–180_	A_181–360_	General (all assessments)	GEE (*p*-values)		
	Groups	Mean (CI 95%)	Mean (CI 95%)	Mean (CI 95%)	Mean (CI 95%)	Mean (CI 95%)	Group	Assessment	Group * assessment
IL-6 (pg/mL)	Moderate COVID (*n* = 22)	2.08 (1.60–2.53)	1.70 (1.42–1.97)	2.18 (1.62–2.74)	1.84 (1.47–2.20)	1.95 (1.69–2.21)^*^	0.004	0.730	0.389
	Severe COVID (*n* = 18)	3.06 (2.33–3.78)	7.69 (−1.35–16.74)	4.35 (0.99–7.71)	3.80 (2.48–5.18)	4.73 (2.44–7.02)^#*^			
	Control (*n* = 30)	1.57 (1.34–1.80)	1.57 (1.34–1.80)	1.57 (1.34–1.80)	1.57 (1.34–1.80)	1.57 (1.34–1.80)			
	Total sample (*n* = 70)	2.24 (1.94–2.53)	3.65 (0.63–6.67)	2.70 (1.56–3.84)	2.41 (1.94–2.88)				
IL-8 (pg/mL)	Moderate COVID (*n* = 22)	4.35 (2.74–5.96)	3.53 (2.71–4.34)	4.73 (2.85–6.61)	4.31 (3.09–5.53)	4.23 (3.05–5.41)	0.017	0.524	0.581
	Severe COVID (*n* = 18)	6.69 (4.80–8.58)	5.98 (4.12–7.85)	5.26 (3.85–6.67)	5.05 (3.61–6.49)	5.75 (4.45–7.04)^*^			
	Control (*n* = 30)	3.77 (3.33–4.21)	3.77 (3.33–4.21)	3.77 (3.33–4.21)	3.77 (3.33–4.21)	3.77 (3.33–4.21)			
	Total sample (*n* = 70)	4.94 (4.10–5.78)	4.43 (3.73–5.12)	4.59 (3.79–5.38)	4.38 (3.73–5.02)				
TNF-α (pg/mL)	Moderate COVID (*n* = 22)	0.98 (0.00–1.96)	1.02 (0.15–1.89)	0.97 (0.36–1.58)	1.01 (0.34–1.67)	0.99 (0.25–1.74)	0.301	0.404	0.017
	Severe COVID (*n* = 18)	0.62 (0.26–0.99)	0.41 (0.14–0.67)	0.16 (0.04–0.37)^a#*^	0.40 (0.13–0.94)	0.40 (0.16–0.63)			
	Control (*n* = 30)	0.53 (0.24–0.82)	0.53 (0.24–0.82)	0.53 (0.24–0.82)	0.53 (0.24–0.82)	0.53 (0.24–0.82)			
	Total sample (*n* = 70)	0.71 (0.35–1.07)	0.65 (0.33–0.97)	0.55 (0.32–0.79)	0.65 (0.34–0.95)				
IL-1β (pg/mL)	Moderate COVID (*n* = 22)	1.00 (0.48–1.52)	0.99 (0.64–1.34)	0.95 (0.51–1.38)	0.77 (0.39–1.15)	0.93 (0.59–1.26)^*^	0.003	0.395	0.213
	Severe COVID (*n* = 18)	1.35 (0.83–1.86)	0.75 (0.34–1.15)	0.86 (0.37–1.36)	1.22 (0.77–1.68)	1.04 (0.74–1.35)^*^			
	Control (*n* = 30)	0.39 (0.13–0.66)	0.39 (0.13–0.66)	0.39 (0.13–0.66)	0.39 (0.13–0.66)	0.39 (0.13–0.66)			
	Total sample (*n* = 70)	0.91 (0.66–1.17)	0.71 (0.51–0.91)	0.73 (0.50–0.97)	0.79 (0.58–1.01)				
IFN-*γ* (pg/mL)	Moderate COVID (*n* = 22)	0.11 (−0.03–0.26)	0.09 (−0.03–0.22)	0.10 (−0.03–0.25)	0.16 (−0.00–0.34)	0.12 (0.04–0.20)	0.262	0.066	0.016
	Severe COVID (*n* = 18)	0.58 (−0.22–0.93)^#*^	0.13 (−0.04–0.31)^a^	0.27 (−0.00–0.55)	0.07 (−0.06–0.21)^a^	0.26 (0.11–0.41)			
	Control (*n* = 30)	0.14 (−0.01–0.29)	0.14 (−0.01–0.29)	0.14 (−0.01–0.29)	0.14 (−0.01–0.29)	0.14 (0.01–0.29)			
	Total sample (*n* = 70)	0.27 (−0.14–0.41)	0.12 (−0.03–0.21)	0.17 (−0.05–0.29)	0.12 (−0.03–0.21)				
IL-12p70 (pg/mL)	Moderate COVID (*n* = 22)	0.62 (0.36–0.87)	0.79 (0.51–1.07)	0.81 (0.52–1.09)	0.54 (0.22–0.85)	0.69 (0.49–0.88)	0.503	0.269	0.057
	Severe COVID (*n* = 18)	0.29 (0.00–0.57)	0.55 (0.22–0.87)	0.56 (0.21–0.90)	0.73 (0.22–1.24)	0.53 (0.33–0.73)			
	Control (*n* = 30)	0.54 (0.22–0.86)	0.54 (0.22–0.86)	0.54 (0.22–0.86)	0.54 (0.22–0.86)	0.54 (0.22–0.86)			
	Total Sample (*n* = 70)	0.48 (0.31–0.65)	0.62 (0.45–0.80)	0.64 (0.45–0.82)	0.60 (0.38–0.83)				
IL-10 (pg/mL)	Moderate COVID (*n* = 22)	1.13 (0.85–1.42)	0.94 (0.63–1.26)	1.24 (0.95–1.53)	1.13 (0.87–1.40)	1.11 (0.90–1.33)	0.648	0.343	0.194
	Severe COVID (*n* = 18)	1.44 (0.94–1.94)	1.20 (0.90–1.49)	1.05 (0.71–1.38)	1.32 (0.96–1.68)	1.25 (1.04–1.46)			
	Control (*n* = 30)	1.22 (0.99–1.44)	1.22 (0.99–1.44)	1.22 (0.99–1.44)	1.22 (0.99–1.44)	1.22 (0.99–1.44)			
	Total Sample (*n* = 70)	1.26 (1.06–1.47)	1.12 (0.96–1.28)	1.17 (1.00–1.33)	1.22 (1.06–1.39)				
Hs_CRP (mg/L)	Moderate COVID (*n* = 22) Severe COVID (*n* = 18) Control (*n* = 30) Total Sample (*n* = 70)	3.64 (1.92–6.91)^*^ 7.39 (4.94–11.06)^*^ 1.08 (0.70–1.64) 3.07 (2.30–4.10)	1.94 (1.18–3.19) 6.46 (4.31–9.69)^#*^ 1.08 (0.70–1.64) 2.38 (1.84–3.08)	2.06 (1.15–3.65) 13.00 (4.29–39.36) 1.08 (0.70–1.64) 3.07 (1.97–4.76)	3.04 (1.84–5.02)^*^ 4.88 (3.39–7.01)^*a^ 1.08 (0.70–1.64) 2.52 (1.96–3.23)	2.58 (1.67–3.98) 7.42 (4.74–11.62) 1.08 (0.70–1.64)	<0.001	0.126	0.012
Hcy (μmol/L)	Moderate COVID (*n* = 22) Severe COVID (*n* = 18) Control (*n* = 30) Total Sample (*n* = 70)	8.99 (6.63–12.22) 9.72 (8.16–11.56) 8.99 (8.09–10.00) 9.23 (8.17–10.43)	9.99 (7.28–13.69)^a^ 13.73 (10.40–18.14)^*a^ 8.99 (8.09–10.00) 10.72 (9.28–12.39)^a^	8.22 (7.10–9.52) 10.90 (8.99–13.20)^#b^ 8.99 (8.09–10.00) 9.30 (8.52–10.16)^b^	7.69 (6.92–8.55)^*^ 10.28 (8.43–12.53)^#b^ 8.99 (8.09–10.00) 8.92 (8.21–9.69)^b^	8.68 (7.18–10.49) 11.06 (9.14–13.38) 8.99 (8.09–10.00)	0.132	<0.001	0.002

A_21–30_: assessment conducted between 21 and 30 days after symptom onset or hospital discharge for severe COVID; A_31–90_: assessment conducted between 31 and 90 days after symptom onset or hospital discharge for severe COVID; A_91–180_: assessment conducted between 91 and 180 days after symptom onset or hospital discharge for severe COVID; and A_181–360_: assessment conducted between 181 and 360 days after symptom onset or hospital discharge for severe COVID. The control group was assessed only once. IL12-p70, Interleukin 12p70 (pg/mL); TNF-α, Tumor Necrosis Factor Alpha (pg/mL); IL-10, Interleukin 10 (pg/mL); IL-6, Interleukin 6 (pg/mL); IL-1β, Interleukin 1 beta (pg/mL); IL-8, Interleukin 8 (pg/mL); IFN-γ, Interferon gamma (pg/mL). Hs_CRP, High-Sensitivity C-Reactive Protein (mg/L); Hcy, Homocysteine (μmol/L). GEE, Generalized estimating equation. *Different from the control group in the respective assessment (*p* < 0.05); #Different from the moderate COVID in the respective assessment (*p* < 0.05); ^a^Different from assessment 1 (*p* < 0.05); ^b^Different from assessment 2 (*p* < 0.05); ^c^Different from assessment 3 (*p* < 0.05).

### Inflammatory markers

We examined additional biomarkers to further investigate the inflammatory and metabolic alterations in individuals with a history of moderate and severe COVID-19. High-sensitivity C-reactive protein (hs-CRP) was assessed as a marker of persistent inflammation, while homocysteine (Hcy) was evaluated due to its role in endothelial dysfunction and cardiovascular risk (Table 3). The analysis of hs-CRP revealed significant differences between groups and assessments, with the severe COVID group showing higher levels than the control and moderate groups across various time points, particularly in A_21–30_, A_31–90_, and A_181–360_. Additionally, severe COVID-19 correlated with greater concentrations of hs-CRP compared to both moderate COVID-19 and control groups, while no effect of assessment over time was observed.

Our analysis also revealed that the severe COVID group had higher levels of Hcy than the control at A_31–90_, and higher levels than the moderate group at A_91–180_. The moderate group showed lower levels than the control at A_181–360_ (Table 3).

### Red blood cells and platelets

Then, we investigated the impact of COVID-19 on hematological parameters to identify possible alterations in oxygen transport mediated by hemoglobin, hematological homeostasis, and platelet-related coagulation mechanisms (Table 4). Red blood cell counts differed across groups and timepoints, with moderate cases showing lower values at A_31–90_ than at later periods, and severe cases presenting fluctuations over time. Hemoglobin levels were reduced in the severe group compared to controls and moderate cases at A_21–30_. In both COVID-19 groups, hemoglobin levels increased progressively over time, particularly from A_21–30_ to A_91–180_ and A_181–360_. Hematocrit followed a similar pattern, with lower values in severe COVID-19 at A_21–30_ compared to controls and higher values at later assessments. Although no association was found for mean corpuscular hemoglobin concentration (MCHC), group differences indicated lower levels in the severe group than in the moderate group. Red cell distribution width (RDW) was consistently elevated in the severe group compared to both moderate and control groups at all timepoints, with significant time-based variation. In contrast, mean corpuscular volume (MCV) and mean corpuscular hemoglobin (MCH) showed no significant differences by group or assessment. Given the role of systemic inflammation in affecting coagulation, platelet count was also evaluated; however, no significant differences were found between groups or across assessments.

**Table 4:** Red blood cell parameters, platelet total count and white blood cell differential count in different disease severities and timepoints of sample collection.

Hemogram	Groups	A_21–30_	A_31–90_	A_91–180_	A_181–360_	General (all assessments)	GEE (*p*-values)		
		Mean (CI 95%)	Mean (CI 95%)	Mean (CI 95%)	Mean (CI 95%)	Mean (CI 95%)	Group	Assessment	Group * assessment
RBC (million/mm^3^)	Moderate COVID (*n* = 22) Severe COVID (*n* = 18) Control (*n* = 30) Total Sample (*n* = 70)	4.83 (4.65–5.02) 4.54 (4.19–4.92) 4.85 (4.67–5.03) 4.74 (4.59–4.89)	4.75 (4.62–4.88) 4.80 (4.48–5.14) 4.85 (4.67–5.03) 4.80 (4.67–4.93)	4.92 (4.78–5.06)^b^ 4.93 (4.60–5.27)^a^ 4.85 (4.67–5.03) 4.90 (4.76–5.03)	4.96 (4.83–5.09)^b^ 4.91 (4.61–5.24)^a^ 4.85 (4.67–5.03) 4.91 (4.78–5.04)	4.86 (4.74–4.99) 4.79 (4.49–5.11) 4.85 (4.67–5.03)	0.915	0.002	0.001
HB (g/dL)	Moderate COVID (*n* = 22) Severe COVID (*n* = 18) Control (*n* = 30) Total Sample (*n* = 70)	14.23 (13.72–14.76) 12.95 (12.03–13.93)^#*^ 14.28 (13.89–14.67) 13.80 (13.41–14.21)	13.96 (13.51–14.41) 13.72 (12.89–14.59)^a^ 14.28 (13.89–14.67) 13.98 (13.64–14.33)	14.46 (13.99–14.94)^b^ 14.05 (13.25–14.90)^a^ 14.28 (13.89–14.67) 14.26 (13.92–14.61)	14.68 (14.25–15.12)^a,b^ 14.03 (13.25–14.85)^a^ 14.28 (13.89–14.67) 14.33 (14.00–14.66)	14.33 (13.91–14.76) 13.68 (12.91–14.49) 14.28 (13.89–14.67)	0.352	<0.001	<0.001
HCT (%)	Moderate COVID (*n* = 22) Severe COVID (*n* = 18) Control (*n* = 30) Total Sample (*n* = 70)	41.64 (39.99–43.36) 38.99 (36.33–41.84)^*^ 42.22 (41.22–43.25) 40.93 (39.78–42.10)	40.36 (39.44–41.97) 40.89 (38.44–43.49) 42.22 (41.22–43.25) 41.26 (40.27–42.28)	42.43 (41.13–43.78)^b^ 41.55 (39.02–44.24)^a^ 42.22 (41.22–43.25) 42.07 (41.04–43.12)	42.64 (41.54–43.77)^b^ 42.12 (39.84–44.54)^a^ 42.22 (41.22–43.25) 42.33 (41.41–43.27)	41.84 (40.64–43.08) 40.87 (38.60–43.28) 42.22 (41.22–43.25)	0.576	<0.001	<0.001
MCV (fL)	Moderate COVID (*n* = 22) Severe COVID (*n* = 18) Control (*n* = 30) Total Sample (*n* = 70)	86.19 (84.39–88.02) 84.24 (78.38–90.53) 85.41 (81.55–89.46) 85.27 (82.80–87.82)	85.55 (84.05–87.08) 85.27 (82.54–88.10) 85.41 (81.55–89.46) 85.41 (83.74–87.12)	86.62 (85.32–87.94)^b^ 85.48 (82.33–88.76) 85.41 (81.55–89.46) 85.84 (84.09–87.62)	86.43 (85.03–87.85)^b^ 86.17 (83.58–88.84) 85.41 (81.55–89.46) 86.00 (84.36–87.68)	85.41 (81.55–89.46) 85.29 (81.96–88.75) 85.41 (81.55–89.46)	0.849	0.181	0.030
MCH (pg)	Moderate COVID (*n* = 22)	29.48 (28.89–30.09)	29.34 (28.87–29.82)	29.36 (28.86–29.87)	29.57 (29.14–30.04)	29.44 (28.98–29.91)	0.523	0.640	0.774
	Severe COVID (*n* = 18)	28.69 (27.15–30.32)	28.71 (27.53–29.94)	28.70 (27.37–30.09)	28.75 (27.60–29.93)	28.71 (27.43–30.04)			
	Control (*n* = 30)	29.56 (28.93–30.19)	29.56 (28.93–30.19)	29.56 (28.93–30.19)	29.56 (28.93–30.19)	29.56 (28.93–30.19)			
	Total sample (*n* = 70)	29.24 (28.64–29.86)	29.20 (28.72–29.69)	29.20 (28.67–29.74)	29.29 (28.82–29.76)				
MCHC (g/dL)	Moderate COVID (*n* = 22)	34.21 (33.74–34.68)	34.32 (33.90–34.74)	33.90 (33.50–34.31)	34.24 (33.85–34.63)	34.17 (33.84–34.49)	0.048	0.245	0.158
	Severe COVID (*n* = 18)	33.20 (32.55–33.85)	33.63 (33.12–34.15)	33.52 (32.93–34.11)	33.32 (32.81–33.83)	33.41 (32.90–33.94)^#^			
	Control (*n* = 30)	33.81 (33.42–34.20)	33.81 (33.42–34.20)	33.81 (33.42–34.20)	33.81 (33.42–34.20)	33.81 (33.42–34.20)			
	Total sample (*n* = 70)	33.74 (33.44–34.04)	33.92 (33.66–34.18)	33.74 (33.47–34.02)	33.79 (33.54–34.04)				
RDW (%)	Moderate COVID (*n* = 22) Severe COVID (*n* = 18) Control (*n* = 30) Total sample (*n* = 70)	12.88 (12.53–13.24) 14.92 (13.99–15.91)^#*^ 12.67 (12.49–12.85) 13.45 (13.14–13.78)	12.99 (12.64–13.35) 14.24 (13.53–14.98)^#*a^ 12.67 (12.49–12.85) 13.28 (13.02–13.55)	13.11 (12.78–13.45)^*^ 14.33 (13.72–14.97)^#*^ 12.67 (12.49–12.85) 13.35 (13.12–13.59)	12.95 (12.55–13.36) 14.01 (13.38–14.67)^#*a,c^ 12.67 (12.49–12.85) 13.20 (12.95–13.45)	12.98 (12.67–13.30) 14.37 (13.71–15.07) 12.67 (12.49–12.85)	<0.001	0.013	0.001
PLT (cell/μL)	Moderate COVID (*n* = 22) Severe COVID (*n* = 18) Control (*n* = 30) Total sample (*n* = 70)	261.75 (233.90–292.91) 270.87 (238.22–307.99) 241.33 (221.49–262.95) 257.68 (241.78–274.63)	265.05 (245.14–286.57) 266.56 (232.32–305.83) 241.33 (221.49–262.95) 257.38 (242.40–273.28)	265.64 (241.10–292.67) 271.35 (231.86–317.58) 241.33 (221.49–262.95) 259.11 (242.10–277.31)	260.18 (238.48–283.86) 260.94 (225.94–301.37) 241.33 (221.49–262.95) 253.99 (238.49–270.50)	263.14 (241.47–286.77) 267.40 (234.43–305.00) 241.33 (221.49–262.95)	0.273	0.304	0.692
WBC (%/mm^3^)	Moderate COVID (*n* = 22) Severe COVID (*n* = 18) Control (*n* = 30) Total sample (*n* = 70)	6375.00 (5908.07–6878.83) 7693.33 (6564.12–9016.79) 5793.33 (5340.20–6284.91) 6574.17 (6162.60–7013.22)	5657.14 (5229.57–6119.66) 7244.44 (6185.74–8484.33) 5793.33 (5340.20–6284.91) 6192.18 (5803.76–6606.59)	6209.09 (5389.98–7152.67) 7635.29 (6346.37–9185.98) 5793.33 (5340.20–6284.91) 6500.21 (5987.17–7057.21)	5968.18 (5489.94–6488.07) 7444.44 (6408.62–8647.68) 5793.33 (5340.20–6284.91) 6361.13 (5970.97–6776.77)	6046.26 (5641.34–6480.23) 7502.3 (6512.46–8642.57)^#*^ 5793.33 (5340.20–6284.91)	0.007	0.121	0.104
BANDS (%/mm^3^)	Moderate COVID (*n* = 22) Severe COVID (*n* = 18) Control (*n* = 30) Total sample (*n* = 70)	0.00 (0.00–0.00) 6.60 (−5.89–19.09) 0.00 (0.00–0.00) 2.20 (−1.96–6.36)	0.00 (0.00–0.00) 0.00 (0.00–0.00) 0.00 (0.00–0.00) 0.00 (0.00–0.00)	13.80 (−12.60–40.22) 0.00 (0.00–0.00) 0.00 (0.00–0.00) 4.60 (−4.20–13.40)	7.61 (−6.95–22.19) 0.00 (0.00–0.00) 0.00 (0.00–0.00) 2.53 (−2.31–7.39)	5.35 (−2.01–12.73) 1.65 (−1.47–4.77) 0.00 (0.00–0.00)	0.212	0.349	0.349
NEU (%/mm^3^)	Moderate COVID (*n* = 22) Severe COVID (*n* = 18) Control (*n* = 30) Total sample (*n* = 70)	3284.69 (2902.95–3716.63) 4522.81 (3714.30–5507.44) 3101.53 (2772.52–3469.59) 3585.05 (3289.48–3907.17)	2914.10 (2613.88–3248.79) 4103.83 (3375.59–4989.19) 3101.53 (2772.52–3469.59) 3334.95 (3068.19–3624.92)	3366.00 (2660.89–4257.95) 4485.12 (3620.81–5555.75) 3101.53 (2772.52–3469.59) 3604.30 (3221.20–4032.98)	3011.68 (2756.57–3290.40) 4120.78 (3407.62–4983.19) 3101.53 (2772.52–3469.59) 3376.41 (3119.17–3654.86)	3138.56 (2867.47–3435.28) 4303.67 (3635.89–5094.10)^#*^ 3101.53 (2772.52–3469.59)	0.003	0.143	0.348
BAS (%/mm^3^)	Moderate COVID (*n* = 22) Severe COVID (*n* = 18) Control (*n* = 30) Total sample (*n* = 70)	48.93 (32.51–65.36) 36.46 (11.66–61.26) 38.33 (27.97–48.69) 41.24 (30.74–51.74)	44.66 (33.31–56.02) 38.94 (19.95–57.92) 38.33 (27.97–48.69) 40.64 (32.50–48.79)	44.85 (31.21–58.49) 32.05 (9.17–54.94) 38.33 (27.97–48.69) 38.41 (28.88–47.94)	51.52 (41.29–61.75) 51.27 (31.61–70.94) 38.33 (27.97–48.69) 47.04 (38.88–55.20)	47.49 (38.39–56.59) 39.68 (20.84–58.53) 38.33 (27.97–48.69)	0.399	0.089	0.156
LYMP (%/mm^3^)	Moderate COVID (*n* = 22) Severe COVID (*n* = 18) Control (*n* = 30) Total sample (*n* = 70)	2278.56 (2060.77–2519.36) 2273.33 (1944.82–2657.33) 2001.53 (1834.89–2183.30) 2180.53 (2036.53–2334.71)	2010.04 (1813.03–2228.46) 2312.16 (2014.24–2654.15) 2001.53 (1834.89–2183.30) 2103.11 (1972.11–2242.81)	1955.86 (1751.45–2184.13) 2331.82 (1953.65–2783.19) 2001.53 (1834.89–2183.30) 2089.93 (1938.31–2253.41)	2095.47 (1817.89–2415.43) 2355.94 (2007.86–2764.36) 2001.53 (1834.89–2183.30) 2145.86 (1986.91–2317.53)	2081.47 (1904.02–2275.46) 2318.11 (2021.85–2657.80) 2001.53 (1834.89–2183.30)	0.206	0.583	0.235
MON (%/mm^3^)	Moderate COVID (*n* = 22) Severe COVID (*n* = 18) Control (*n* = 30) Total sample (*n* = 70)	638.06 (485.87–837.91)^*^ 645.86 (540.82–771.31)* 438.83 (409.96–469.73) 565.50 (506.21–631.73)	553.95 (461.24–665.29)^*^ 587.88 (487.95–708.28)* 438.83 (409.96–469.73) 522.82 (477.82–572.05)	559.90 (487.69–642.81)^*^ 625.94 (510.25–767.86)* 438.83 (409.96–469.73) 535.77 (491.97–583.47)	551.98 (412.45–738.71)^a^ 576.11 (489.09–678.60)^*^ 438.83 (409.96–469.73) 518.69 (462.94–581.15)	574.90 (465.37–710.21) 608.30 (513.42–720.70) 438.83 (409.96–469.73)	<0.001	0.006	0.003

A_21–30_: assessment conducted between 21 and 30 days after symptom onset or hospital discharge for severe COVID; A_31–90_: assessment conducted between 31 and 90 days after symptom onset or hospital discharge for severe COVID; A_91–180_: assessment conducted between 91 and 180 days after symptom onset or hospital discharge for severe COVID; and A_181–360_: assessment conducted between 181 and 360 days after symptom onset or hospital discharge for severe COVID. The control group was assessed only once. RBC, Red Blood Cell Count (million/mm^3^); HB, Hemoglobin (g/dL); HCT, Hematocrit (%); MCV, Mean Corpuscular Volume (fL); MCH, Mean Corpuscular Hemoglobin (pg); MCHC, Mean Corpuscular Hemoglobin Concentration (g/dL); RDW, Red Cell Distribution Width (%); PLT, Platelet Count (cell/μL). WBC, White Blood Cell Count (%/mm^3^); BANDS, Band neutrophils ++s (%/mm^3^); NEU, Neutrophils (%/mm^3^); BAS, Basophils (%/mm^3^); LYMP, Lymphocytes (%/mm^3^); MON, Monocytes (%/mm^3^). GEE, Generalized estimating equation. ^*^Different from the control group in the respective assessment (*p* < 0.05); ^#^Different from the moderate COVID in the respective assessment (*p* < 0.05); ^a^Different from assessment 1 (*p* < 0.05); ^b^Different from assessment 2 (*p* < 0.05); ^c^Different from assessment 3 (*p* < 0.05).

### White blood cells

We assessed leukocyte profiles to investigate immune alterations (Table 4). White blood cell counts were elevated in the severe group compared to moderate and control groups, despite no significant differences across timepoints. Similarly, neutrophil levels were higher in severe cases than in moderate and control groups, without time-based variation. Monocytes showed both group and time point effects, with severe cases consistently presenting higher levels than controls, and moderate cases showing transient elevations. Monocyte levels were highest at A_21–30_ and decreased over time. No significant differences were observed for band cells, basophils, or lymphocytes across groups or assessments.

### Hematological indices

**Figure 2:**
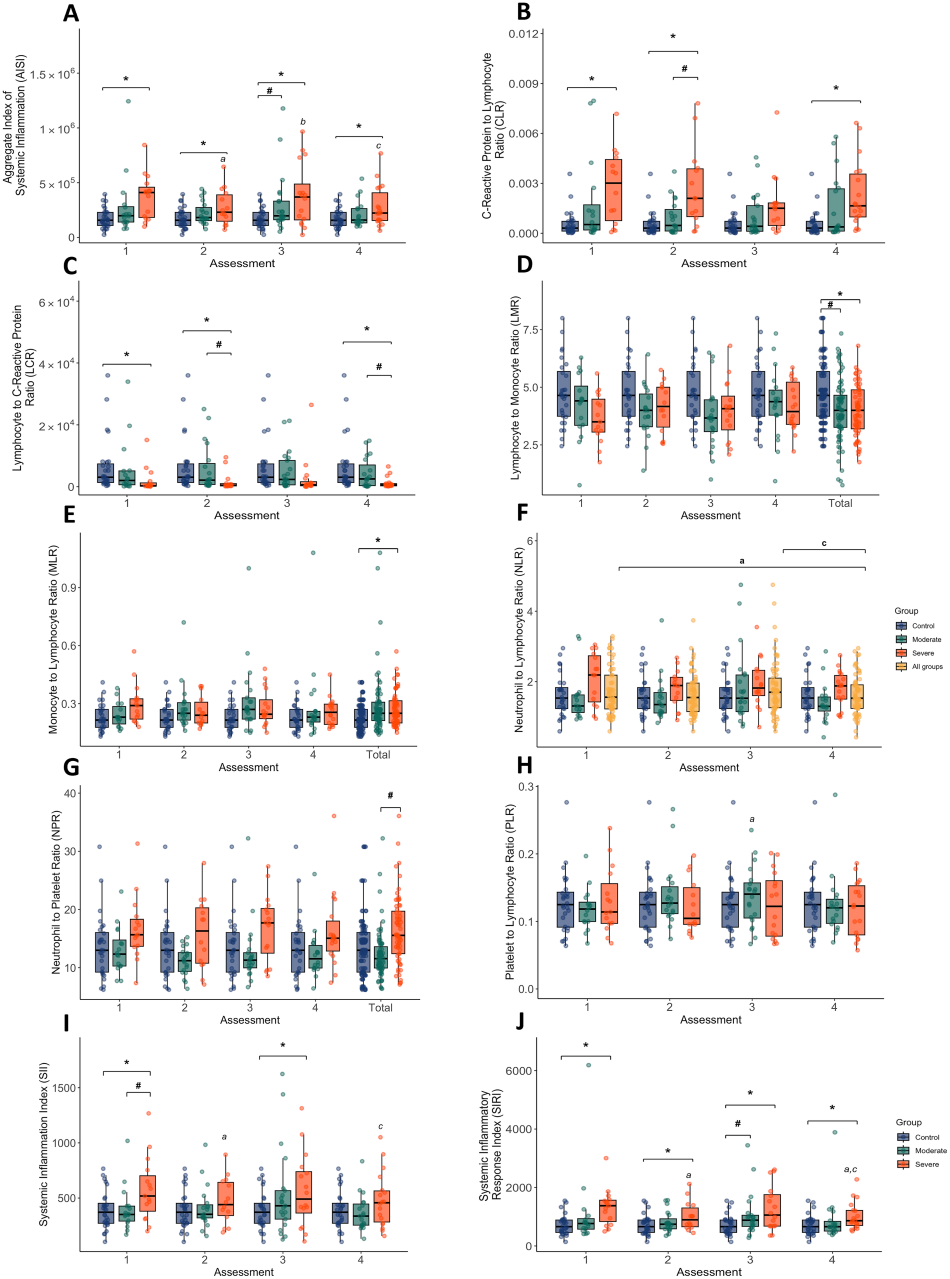
Hematological indices in different disease severities and timepoints of sample collection. Comparison of **(A)** aggregate index of systemic inflammation (AISI), **(B)** C-reactive protein to lymphocyte ratio (CLR), **(C) l**ymphocyte to C-reactive protein ratio (LCR), **(D)** lymphocyte to monocyte ratio (LMR), **(E)** monocyte to lymphocyte ratio (MLR), **(F)** neutrophil to lymphocyte ratio (NLR), **(G)** neutrophil to platelet ratio (NPR), **(H)** platelet to lymphocyte ratio (PLR), **(I)** systemic immune-inflammatory (SII), and **(J)** systemic inflammation response index (SIRI) in control, moderate, and severe groups across different assessments. *Difference between groups in the same assessment a: Different from assessment 1 (A_21–30_), b: Different from assessment 2 (A_31–90_), c: Different from assessment 3 (A_91–180_). Measurements were conducted at various assessment time points, as indicated in the assessment axis. Each point in the graph represents a patient’s value within each group and assessment period. Vertical bars represent the mean and standard deviation for each condition. Groups were represented as it follows: control in blue, moderate COVD-19 in green and severe COVID-19 in orange. The graphs were generated using RStudio (RStudio, PBC, Boston, MA, United States). Additionally, hematological index trajectories for the ontrol, oderate COVID-19, and ever COVID-19 groups across the four assessment periods are provided in Supplementary material (in this figure). #Difference compared to moderate COVID-19 group.

We also evaluated hematological indices to further understand the systemic impact of COVID-19 (Figure 2 and Supplementary Table 1). AISI levels were consistently elevated in severe cases compared to controls across all assessments and showed time-based variation within the severe group (Figure 2A). CLR was also increased in severe cases compared to controls and moderate cases at specific timepoints (Figure 2B). Conversely, LCR was reduced in severe COVID-19 compared to controls and moderate cases (Figure 2C). Although LMR showed no discrepancies, severe and moderate groups exhibited lower levels than controls participants (Figure 2D). On the other hand, MLR showed higher levels in severe cases compared to controls (Figure 2E). NLR varied over time, with higher levels at earlier assessments, but no group differences were found (Figure 2F). NPR revealed increased levels in the severe group compared to moderate, while PLR showed time-based variation in moderate cases without clear group differences (Figures 2G,H). SII was higher in severe cases at selected timepoints and varied across assessments (Figure 2I). SIRI was consistently elevated in severe COVID-19 and demonstrated significant temporal variation (Figure 2J). MNR, NLPR, and RPR showed no significant differences by group or assessment.

### Kidney function and skeletal muscle and liver damage

We investigated markers of renal function and hepatic and skeletal muscle damage (Table 5). Creatinine levels varied over time, with severe cases showing lower values at A_21–30_ compared to later assessments. Aspartate aminotransferase (AST) levels were higher at A_31–90_ than at A_91–180_ in severe cases, with time-based differences but no consistent group effect. Alkaline phosphatase (ALP) showed no group differences, though levels increased from A_91–180_ to A_181–360_. Gamma-glutamyl transferase (GGT) levels were consistently elevated in the severe group compared to moderate and control groups across several timepoints, indicating persistent hepatic stress. Ferritin levels were higher in the severe group at A_21–30_ compared to later periods, suggesting residual inflammation, despite no sustained group effect. Additionally, creatine phosphokinase (CPK) was evaluated due to symptoms like muscle weakness, with severe cases presenting lower levels at A_21–30_ compared to later assessments. Overall, time-dependent improvements were observed across markers, though severe cases demonstrated more pronounced alterations in hepatic and muscle-related parameters.

**Table 5:** Markers of kidney function, skeletal muscle and liver damage and lipid profile in different disease severities and timepoints of sample collection.

Hemogram	Groups	A_21–30_	A_31–90_	A_91–180_	A_181–360_	General (all assessments)	GEE (*p*-values)		
		Mean (CI 95%)	Mean (CI 95%)	Mean (CI 95%)	Mean (CI 95%)	Mean (CI 95%)	Group	Assessment	Group * assessment
CREA (mg/dL)	Moderate COVID (*n* = 22) Severe COVID (*n* = 18) Control (*n* = 30) Total sample (*n* = 70)	0.79 (0.70–0.88) 0.85 (0.69–1.04) 0.85 (0.80–0.92) 0.83 (0.76–0.90)	0.81 (0.74–0.88) 0.90 (0.76–1.07)^a^ 0.85 (0.80–0.92) 0.85 (0.80–0.91)	0.82 (0.75–0.89) 0.93 (0.77–1.11) ^a^ 0.85 (0.80–0.92) 0.86 (0.81–0.93)	0.83 (0.77–0.90) 0.87 (0.76–1.01) 0.85 (0.80–0.92) 0.85 (0.80–0.91)	0.81 (0.74–0.88) 0.89 (0.75–1.05) 0.85 (0.80–0.92)	0.538	0.053	0.005
AST (U/L)	Moderate COVID (*n* = 22) Severe COVID (*n* = 18) Control (*n* = 30) Total sample (*n* = 70)	20.81 (19.06–22.73) 25.73 (18.23–36.30) 20.48 (18.74–22.38) 22.22 (19.66–25.10)	24.40 (20.03–29.71) 24.66 (20.33–29.92) 20.48 (18.74–22.38) 23.10 (20.97–25.44)	22.09 (19.60–24.90) 19.47 (16.57–22.87)^b^ 20.48 (18.74–22.38) 20.65 (19.19–22.22) ^b^	23.00 (20.26–26.10) 21.05 (17.47–25.36) 20.48 (18.74–22.38) 21.48 (19.82–23.29)	22.54 (20.40–24.90) 22.58 (19.44–26.23) 20.48 (18.74–22.38)	0.299	0.034	0.012
ALT (U/L)	Moderate COVID (*n* = 22) Severe COVID (*n* = 18) Control (*n* = 30) Total sample (*n* = 70)	21.954 (18.43–26.14) 25.73 (20.21–32.75) 19.41 (16.91–22.28) 22.21 (19.91–24.78)	25.65 (19.51–33.71) 23.77 (19.11–29.57) 19.41 (16.91–22.28) 22.79 (20.10–25.83)	22.38 (18.48–27.10) 19.52 (16.07–23.72) 19.41 (16.91–22.28) 20.39 (18.42–22.58)^b^	24.27 (19.70–29.90) 20.72 (17.32–24.77) 19.41 (16.91–22.28) 21.37 (19.29–23.68)	23.51 (19.46–28.42) 22.30 (19.52–25.48) 19.41 (16.91–22.28)	0.197	0.043	0.062
ALP (U/L)	Moderate COVID (*n* = 22) Severe COVID (*n* = 18) Control (*n* = 30) Total sample (*n* = 70)	68.77 (60.71–77.90) 77.60 (68.09–88.42) 67.00 (59.22–75.79) 70.97 (65.98–76.34)	66.05 (57.31–76.11) 78.00 (70.71–86.03) 67.00 (59.22–75.79) 70.14 (65.36–75.28)	67.38 (60.27–75.32) 73.17 (60.74–88.15) 67.00 (59.22–75.79) 69.12 (63.31–75.12)	71.09 (64.26–78.63) 83.50 (70.32–99.14) 67.00 (59.22–75.79) 73.54 (68.01–79.51)^c^	68.29 (61.11–76.32) 77.98 (68.83–88.35) 67.00 (59.22–75.79)	0.176	0.034	0.130
GGT (U/L)	Moderate COVID (*n* = 22) Severe COVID (*n* = 18) Control (*n* = 30) Total sample (*n* = 70)	23.77 (20.27–27.87) 55.26 (38.98–78.34)^#*^ 25.68 (18.47–35.72) 32.31 (27.30–38.25)	20.95 (17.19–25.52) 47.33 (31.89–70.23)^#*^ 25.68 (18.47–35.72) 29.42 (24.48–35.35)	20.66 (16.58–25.75) 40.52 (26.45–62.09)^#^ 25.68 (18.47–35.72) 27.81 (22.90–33.77)	23.81 (18.56–30.56) ^c^ 50.05 (30.81–81.32)^#^ 25.68 (18.47–35.72) 31.28 (25.29–38.69)	22.25 (18.47–26.79) 47.99 (35.29–65.27) 25.68 (18.47–35.72)	<0.001	0.148	0.023
FT (ng/dL)	Moderate COVID (*n* = 22) Severe COVID (*n* = 18) Control (*n* = 30) Total sample (*n* = 70)	143.32 (102.19–201.00) 253.39 (158.77–404.38) 144.46 (110.71–188.50) 173.76 (140.59–214.75)	138.28 (90.69–210.83) 187.39 (110.10–318.94)^a^ 144.46 (110.71–188.50) 155.27 (121.77–197.98) ^a^	137.45 (88.76–212.85) 148.09 (88.96–246.51)^a^ 144.46 (110.71–188.50) 143.26 (112.61–182.26) ^a^	135.87 (90.86–203.18) 313.98 (132.28–745.28) 144.46 (110.71–188.50) 183.34 (131.81–255.02)	138.70 (94.40–203.78) 216.77 (129.09–363.99) 144.46 (110.71–188.50)	0.340	0.005	0.010
CPK (U/L)	Moderate COVID (*n* = 22) Severe COVID (*n* = 18) Control (*n* = 30) Total sample (*n* = 70)	95.18 (68.45–132.35) 73.66 (49.35–109.96)^*^ 136.13 (107.70–172.05) 98.46 (81.44–119.03)	222.90 (112.27–44.55) 105.50 (78.36–142.04)^a^ 136.13 (107.70–172.05) 147.38 (113.51–191.35) ^a^	121.18 (85.04–172.66) 112.32 (79.96–157.80)^a^ 136.13 (107.70–172.05) 122.82 (102.46–147.24) ^a^	186.54 (110.64–314.58)^a^ 102.88 (76.87–137.71)^a^ 136.13 (107.70–172.05) 137.73 (111.18–170.62)^a^	147.98 (104.22–210.13) 97.35 (72.50–130.71) 136.13 (107.70–172.05)	0.125	<0.001	<0.001
TG (mg/dL)	Moderate COVID (*n* = 22)Severe COVID (*n* = 18)Control (*n* = 30)Total sample (*n* = 70)	114.43 (88.35–148.21)182.40 (153.64–216.53)106.86 (91.00–125.49)130.66 (116.29–146.80)	95.57 (79.49–114.90)155.88 (127.26–190.95)106.86 (91.00–125.49)116.76 (105.03–129.81)^a^	108.81 (85.169–139.03)155.47 (127.44–189.65)106.86 (91.00–125.49)121.82 (108.26–137.08)	118.13 (89.76–155.47)162.27 (127.64–206.30)106.86 (91.00–125.49)127.00 (111.20–145.05)	108.89 (87.09–136.14)163.65 (138.96–192.74)^#*^106.86 (91.00–125.49)	<0.001	0.019	0.086
COLT (mg/dL)	Moderate COVID (*n* = 22) Severe COVID (*n* = 18) Control (*n* = 30) Total sample (*n* = 70)	189.75 (174.59–206.22) 183.26 (165.64–202.76) 178.36 (165.47–192.26) 183.73 (174.72–193.26)	181.52 (168.28–195.81) 175.61 (158.65–194.38) 178.36 (165.47–192.26) 178.48 (169.93–187.46)	184.40 (170.48–199.45) 179.29 (160.61–200.15) 178.36 (165.47–192.26) 180.67 (171.59–190.22)	184.18 (168.91–200.83) 177.88 (160.26–197.45) 178.36 (165.47–192.26) 180.12 (171.05–189.67)	184.94 (172.07–198.77) 178.99 (163.54–195.90) 178.36 (165.47–192.26)	0.760	0.427	0.696
HDL (mg/dL)	Moderate COVID (*n* = 22) Severe COVID (*n* = 18) Control (*n* = 30) Total sample (*n* = 70)	50.68 (46.39–55.37) 41.93 (36.38–48.33) 49.30 (45.39–53.54) 47.14 (44.30–50.17)	51.61 (46.19–57.67) 44.33 (38.62–50.88) 49.30 (45.39–53.54) 48.32 (45.27–51.57)	50.22 (46.02–54.81) 45.88 (39.08–53.86) 49.30 (45.39–53.54) 48.43 (45.30–51.77)	53.04 (47.14–59.68) 46.22 (40.75–52.42) 49.30 (45.39–53.54) 49.44 (46.39–52.70)	51.38 (46.98–56.19) 44.56 (39.14–50.72) 49.30 (45.39–53.54)	0.207	0.175	0.230
LDL (mg/dL)	Moderate COVID (*n* = 22) Severe COVID (*n* = 18) Control (*n* = 30) Total sample (*n* = 70)	123.37 (110.43–137.82) 113.93 (99.31–130.70) 112.06 (100.62–124.81) 116.35 (108.60–124.65)	115.33 (104.08–127.80) 109.00 (95.53–124.36) 112.06 (100.62–124.81) 112.10 (104.91–119.78)	118.13 (105.48–132.30) 113.17 (97.43–131.45) 112.06 (100.62–124.81) 114.43 (106.46–122.99)	115.77 (103.44–129.56) 111.55 (97.27–127.92) 112.06 (100.62–124.81) 113.11 (105.55–121.21)	118.11 (106.99–130.38) 111.90 (98.80–126.72) 112.06 (100.62–124.81)	0.718	0.428	0.474
N_HDL (mg/dL)	Moderate COVID (*n* = 22) Severe COVID (*n* = 18) Control (*n* = 30) Total sample (*n* = 70)	139.06 (124.54–155.27) 139.33 (121.92–159.23) 129.06 (116.80–142.61) 135.73 (126.98–145.08)	129.90 (117.39–143.74) 131.27 (115.57–149.11) 129.067 (116.80–142.61) 130.08 (122.06–138.62)	132.22 (118.54–147.49) 133.41 (115.89–153.58) 129.067 (116.80–142.61) 131.55 (122.89–140.82)	131.13 (117.35–146.54) 131.66 (114.84–150.95) 129.067 (116.80–142.61) 130.61 (122.09–139.73)	133.03 (120.73–146.59) 133.88 (118.89–150.76) 129.067 (116.80–142.61)	0.873	0.214	0.274

A_21–30_: assessment conducted between 21 and 30 days after symptom onset or hospital discharge for severe COVID; A_31–90_: assessment conducted between 31 and 90 days after symptom onset or hospital discharge for severe COVID; A_91–180_: assessment conducted between 91 and 180 days after symptom onset or hospital discharge for severe COVID; and A_181–360_: assessment conducted between 181 and 360 days after symptom onset or hospital discharge for severe COVID. The control group was assessed only once. CREA, Creatinine (mg/dL); AST, Aspartate Aminotransferase (U/L); ALT, Alanine Aminotransferase (U/L); ALP, Alkaline Phosphatase (U/L); GGT, Gamma-Glutamyl Transferase (U/L); FT, Ferritin (ng/dL); CPK, Creatine Phosphokinase (U/L); TG, Triglycerides (mg/dL); COLT, Cholesterol (mg/dL); HDL, High-Density Lipoprotein (mg/dL); LDL, Low-Density Lipoprotein (mg/dL); N_HDL, Non-High-Density Lipoprotein (mg/dL); GEE, Generalized estimating equation. *Different from the control group in the respective assessment (*p* < 0.05); #Different from the moderate COVID in the respective assessment (*p* < 0.05); ^a^Different from assessment 1 (*p* < 0.05); ^b^Different from assessment 2 (*p* < 0.05); ^c^Different from assessment 3 (*p* < 0.05).

### Lipid profile

We assessed lipid metabolism to explore long-term metabolic disturbances in participants with a history of moderate or severe acute COVID-19 (Table 5). Triglyceride levels were elevated in the severe group compared to moderate and control groups, with higher concentrations observed at A_21–30_ compared to A_31–90_. In contrast, total cholesterol, high-density lipoprotein (HDL), and non-HDL cholesterol showed no significant differences across groups or timepoints.

### MMPs levels and activity

**Figure 3:**
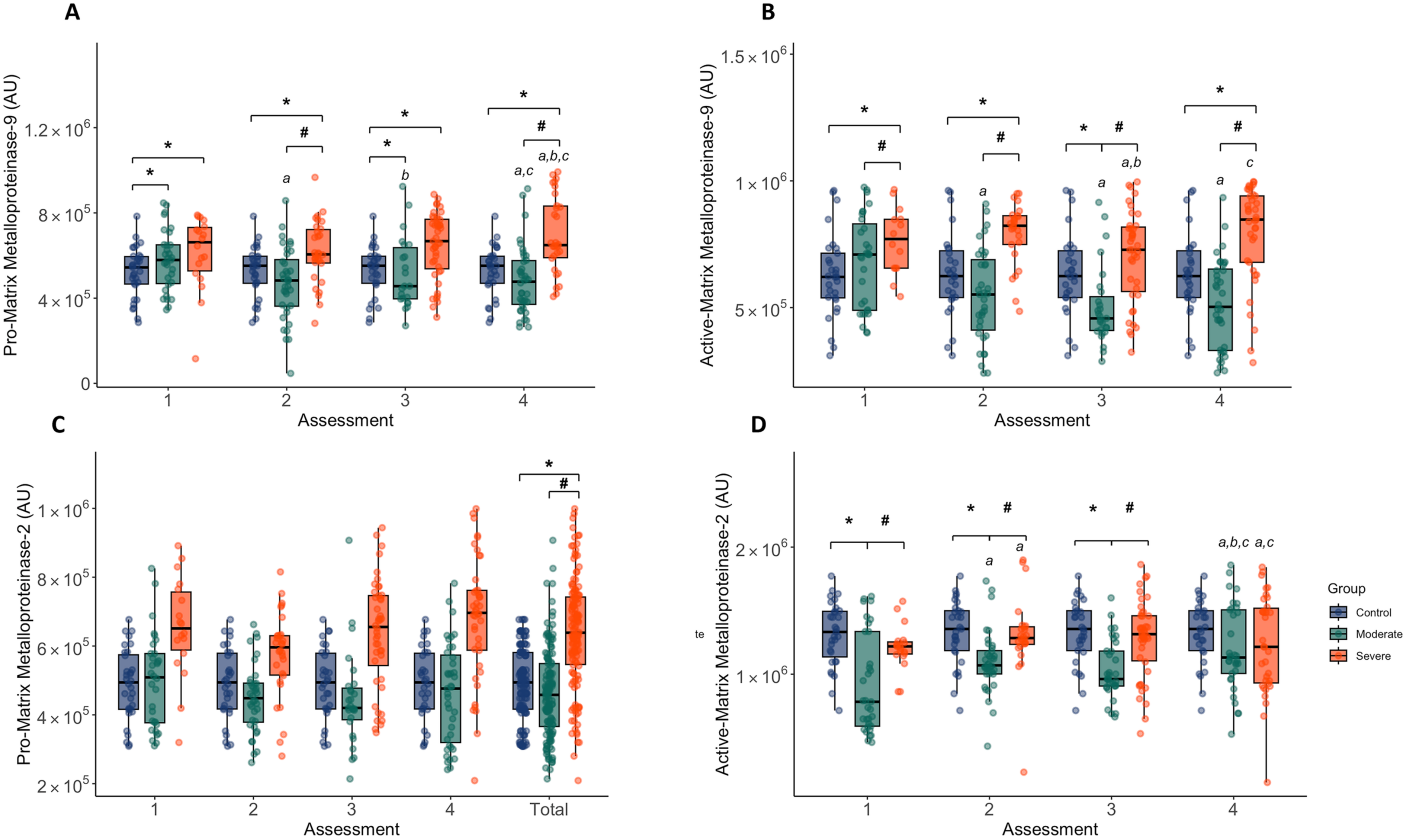
Gelatinase levels activity in different disease severity and timepoints of sample collection. Comparison of **(A)** Pro MMP-9 levels, **(B)** Active MMP-9 levels, **(C)** Pro MMP-2 levels, and **(D)** Active MMP-9 levels in control participants, and in moderate and severe COVID-19 groups across different assessments. *Difference between groups in the same assessment. ^a^Different from assessment 1 (A_21–30_), ^b^Different from assessment 2 (A_31–90_), ^c^Different from assessment 3 (A_91–180_). Measurements were conducted at various assessment time points, as indicated in the assessment axis. Each point in the graph represents a patient’s value within each group and assessment period. Vertical bars represent the mean and standard deviation for each condition. Groups were represented as it follows: control in blue, moderate COVID-19 in green and severe COVID-19 in orange. The graphs were generated using RStudio (RStudio, PBC, Boston, MA, United States). Additionally, gelatinase activity trajectories for the control, moderate COVID-19, and severe COVID-19 groups across the four assessment periods are provided in Supplementary material (in this figure). #Difference compared to moderate COVID-19 group.

We focused on MMPs levels and activity due to their role in tissue remodeling and inflammation (Figure 3 and Supplementary Table 2). Pro-MMP-2 levels were higher in the severe group compared to moderate and control groups, without significant differences across timepoints. Active MMP-2 varied across both groups and assessments, with moderate cases showing lower levels than controls and severe cases early on, followed by progressive increases over time. Pro-MMP-9 levels were elevated in both moderate and severe groups compared to controls, especially at early timepoints, with severe cases maintaining higher levels across the study. Active MMP-9 was consistently increased in the severe group compared to both control and moderate groups at all timepoints, with time-based variations observed particularly within moderate cases. Overall, MMP alterations highlighted persistent matrix remodeling activity, especially in individuals who had experienced severe acute COVID-19.

## Discussion

This longitudinal study characterizes hematological parameters of individuals with prior severe acute COVID-19 over 12 months after symptom onset. The control group was assessed at a single time point, which limits direct comparison of longitudinal trajectories. Additionally, prior asymptomatic SARS-CoV-2 infection cannot be entirely excluded. Therefore, the control group should be interpreted as a cross-sectional reference rather than a true longitudinal comparator. Our findings should be interpreted as reflecting persistent biological alterations following COVID-19 rather than direct measures of clinically defined long COVID-19 syndromes. Renal, hepatic, and muscle injury markers were largely stable. In contrast, the severe group showed an unfavorable inflammatory and cardiometabolic profile with higher triglycerides, increased homocysteine, persistent immune dysregulation, and heightened systemic inflammation. These results agree with our serial high-sensitivity C-reactive protein and homocysteine patterns and are consistent with reports of low-grade systemic inflammation in cohorts with persistent post-acute symptoms after severe COVID-19.

**Figure 4:**
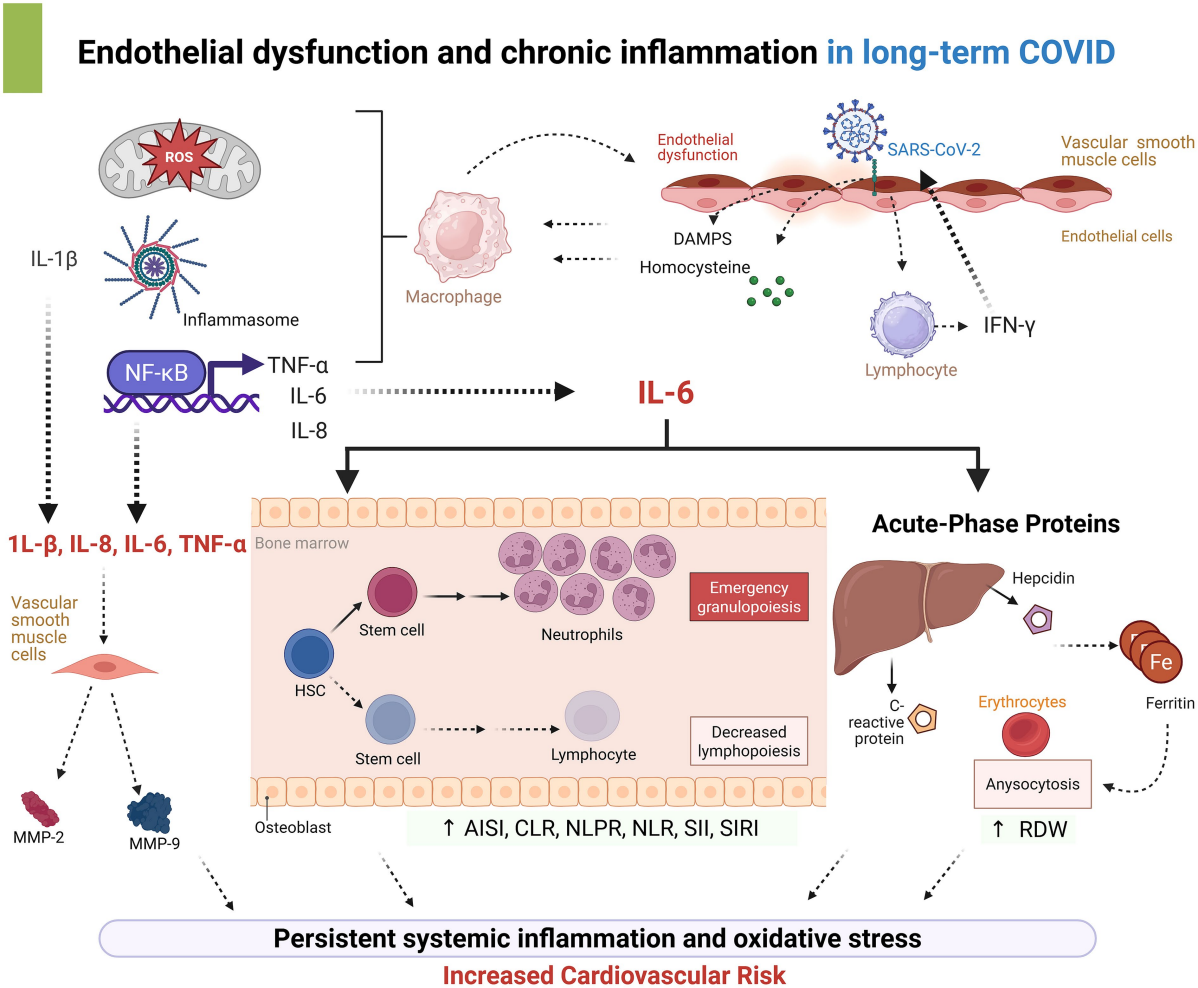
Endothelial dysfunction and chronic inflammation in post-acute COVID consequences. SARS-CoV-2 infection triggers a plethora of pathological alterations that lead to persistent inflammatory responses, hematological derangements, and endothelial dysfunction. *AISI,* Aggregate Index of Systemic Inflammation; *CLR,* C-Reactive Protein to Lymphocyte Ratio; IFN, Interferon; IL, Interleukin; MMP, Matrix metalloproteinase; NF-κB, Nuclear Factor κB; *NLPR,* Neutrophil to Lymphocyte and Platelet Ratio; *NLR,* Neutrophil to Lymphocyte Ratio; RDW, *Red cell distribution width;* ROS, Reactive oxygen species; *SII,* Systemic Inflammation Index; *SIRI,* Systemic Inflammatory Response Index; TNF, Tumor Necrosis Factor. Created in BioRender. Moreno (2026) https://BioRender.com/0flw9au. License (Agreement number):TS29SIQ8RW.

Accumulating clinical evidence suggests that the pathophysiology of severe SARS-CoV-2 infection is largely characterized by endothelial dysfunction. However, it remains unclear whether the virus directly or indirectly contributes to the development of this dysfunction in COVID-19 sequelae. It is widely recognized that severe clinical manifestations of COVID-19 can trigger cytokine storm syndrome and a systemic pro-inflammatory response, potentially leading to endotheliitis and endothelial dysfunction (Figure 4) (47). This phenomenon depends on the entry of SARS-CoV-2 into endothelial cells, which is mediated by Angiotensin-converting enzyme 2 (ACE2) expression. In line with these conclusions, more pronounced characteristics of endotheliitis have been observed in capillaries with higher ACE2 expression compared to major coronary arteries, where ACE2 is expressed at minimal levels (48). Furthermore, *in vitro* studies indicate that senescent endothelial cells exhibit greater functional deterioration upon viral infection, which may partially explain the age-related cardiovascular complications and sequelae associated with SARS-CoV-2 infection (49).

Mechanistically, endothelial activation and inflammatory cascades promote leukocyte adhesion, endothelial permeability, reactive oxygen species, and expression of IL-6, IL-8, TNF-*α*, and IFN-*γ* (50). This sustained inflammatory and oxidative milieu perpetuates endothelial injury (51) and increases neurologic, cardiovascular, autoimmune, pulmonary, inflammatory, and metabolic risks (52). Our severe group maintained higher IL-1β, IL-6, and IL-8 levels, with persistently elevated high-sensitivity C-reactive protein and no compensatory rise in IL-10. IL-1β was consistently higher in severe cases, which agrees with reports of elevation up to 8 months and even 2 years after severe disease (5, 37), and it is linked to tissue injury and sustained inflammation (53, 54). IFN-γ remained higher at three to 4 weeks in severe cases, consistent with its association with post-acute sequelae and fibrotic complications (55, 56). IL-8 showed intergroup differences, in agreement with persistent elevations reported up to 8 months (57) and with data suggesting aberrant dynamics that impair tissue repair (58). The IL-6 axis was coherent in our cohort, since severe cases had higher C-reactive protein and ferritin (59). Increased C-reactive protein has been described at 6 weeks, 2 months, and 6 months (4, 60, 61), and it associates with both acute disease severity and post-acute sequelae of COVID-19 (62).

Hematological findings mirrored these inflammatory responses. RDW remained elevated for 12 months in severe cases, echoing persistent abnormalities reported up to 985 days (63). Elevated RDW reflects anisocytosis and may result from hepcidin-driven iron sequestration (64). It rises within 15–30 days after infection and correlates with mortality (65–67). In our data, sustained RDW tracked with CRP, supporting its value as a dual biomarker of acute severity and long-term risk (68). IL-6 promotes myelopoiesis and impairs lymphopoiesis (69), and higher levels of neutrophils and monocytes with lymphopenia correlate with severity and mortality (70). Several studies suggest that augmented hematological indices, including AISI, CLR, NLPR, NLR, SII, and SIRI, correlate with increased severity of acute COVID-19 (14–18, 20). Lionte and colleagues identified increased SII, NLR, and MLR as markers of severe disease up to 30 days post-infection (71). Furthermore, Gennaro and collaborators demonstrated a direct association between hematological indices and long-term COVID-19 complications, reporting that SII remained elevated in patients 3 months after disease onset (72). To the best of our knowledge, we provide the first evidence that several hematological indices can remain disrupted in severe COVID-19 cases even 1 year after the acute disease. Specifically, AISI, CLR, NPR, and SIRI were significantly elevated in severe COVID-19 patients at the 1-year mark, while SII peaked in critical cases within 2 weeks and remained elevated in moderate cases for up to 6 months. Our follow-up study highlights the potential of the accessible hematological indices as valuable tools for assessing the occurrence of COVID-19-persistent alterations as distant as far as 1-year post-disease onset. Although certain biomarkers demonstrated persistent alterations, this study does not establish predictive models or clinical risk stratification tools, and no conclusions regarding predictive performance can be drawn.

MMPs were also associated with individual inflammatory status (73). Matrix metalloproteinases are known to contribute to extracellular matrix degradation, endothelial dysfunction, and vascular remodeling. Emerging evidence also suggests their involvement in mitochondrial dysfunction through oxidative stress and inflammatory signaling pathways. Thus, the altered MMP profile observed in our cohort may reflect not only systemic inflammation but also underlying vascular and mitochondrial disturbances linked to long-term manifestations associated with severe acute COVID-19 (74, 75). Active MMP-2 upregulation in severe COVID-19 is linked to tissue damage, renin-angiotensin-aldosterone system (RAAS) hyperactivation, prolonged inflammation, and disrupted MMP/TIMP (tissue inhibitor of metalloproteinases) balance, perpetuating inflammatory cascades, endothelial dysfunction, and cardiovascular involvement (76). Concurrently, MMP-9 drives extracellular matrix (ECM) remodeling and neutrophil-mediated inflammation, exacerbating pulmonary injury and systemic complications (29). Cavalcante and collaborators have reported elevated levels of MMPs in serum samples of COVID-19 patients after 2 weeks and 1 month after disease onset (26). Increased MMP levels were also connected to the occurrence of long-term COVID-19 complications, as Lerum and colleagues stated that MMP-9 levels were detected in elevated amounts in patients after 3 months of disease onset (27). Zingaropoli and others also verified increased MMP-9 levels in patients exhibiting persistent alterations due to COVID-19, along with augmented MMP-2 activity, also 3 months after disease onset (28). Herein, we showed that the levels and activity of MMP-2 and MMP-9 are augmented in plasma samples of patients affected by severe COVID-19, even after 1 year of infection. Our data highlight that circulating levels and activity of MMP-2 and MMP-9 are strongly associated with prior severe COVID-19 and with prolonged post-acute biomarker abnormalities after infection Inflammation and matrix metalloproteinases reinforce each other. Tumor necrosis factor alpha (TNF-*α*) induces MMP-9 (77, 78), whereas IL-1β increases MMP-2 and MMP-9 in cells and animal models and in diabetic patients (79–81). IL-6 and IL-8 stimulate matrix metalloproteinase secretion and MMP-9 knockout reveals interdependence with the IL-6 axis (82–84). Neutrophils are a major source of MMP-9 in acute COVID-19 (85), and oxidative stress activates MMP-2 and MMP-9 (86). Once activated, these enzymes drive proteolytic shedding of receptors and process pro-IL-1β (87, 88). Matrix degradation increases mediator bioavailability and sustains inflammation (89). Imbalance with tissue inhibitors has been reported in COVID-19 and relates to persistent injury including acute respiratory distress syndrome (90). MMP-2 and MMP-9 predict severity and mortality (91). Our data indicate that their alterations may also be associated with an increased risk of persistent abnormalities up to 1 year after infection (91, 92).

This study has several limitations that should be considered when interpreting the findings. Limitations of our work include baseline imbalances such as body mass index, and absence of pre-infection clinical data, with possible confounding from comorbidities and physical activity. Even so, our findings are aligned with published literature and are unlikely to reflect anthropometric differences alone. An important limitation of the present study is that the control group was assessed only once, whereas the COVID-19 groups were followed longitudinally. Thus, the control group should be interpreted as a cross-sectional reference rather than a true longitudinal comparator. In addition, because controls were defined as individuals who tested negative or remained asymptomatic during the pandemic, prior asymptomatic or unrecognized SARS-CoV-2 infection cannot be completely excluded. No sensitivity analyses were performed to specifically address this issue. Therefore, caution is warranted when interpreting the observed differences as persistent or exclusively COVID-specific, since some alterations may also reflect normal temporal variation, recovery from severe illness, hospitalization, or baseline differences in clinical characteristics. Another important limitation is that the temporal origin of follow-up differed according to clinical presentation, being referenced to symptom onset in non-hospitalized moderate cases and to hospital discharge in severe hospitalized cases. Although this approach was adopted to reflect the first feasible post-acute timepoint in each subgroup, these anchors are not biologically equivalent and may capture different stages of recovery. In addition, the primary GEE models were not adjusted for baseline clinical covariates such as BMI, comorbidities, smoking history, vaccination status, or hospitalization-related factors. Therefore, caution is warranted when interpreting the observed biomarker abnormalities as mechanisms specific to long COVID-19 syndromes, since some findings may also reflect the lingering effects of severe acute illness, hospitalization, or differences in baseline clinical profile.

In summary, chronic inflammation and immune dysregulation persisted over 1 year, particularly after severe acute disease. We report that changes in hematological indices and matrix metalloproteinases at 1 year are associated with a history of severe acute COVID-19. This integrated panel aligns with cytokines and other inflammatory markers across 12 months (4–6, 14–20, 26–30, 37, 59–62, 68, 72, 73, 76, 82–92) and supports future multicenter validation and therapeutic studies that target matrix metalloproteinase activity and correct immune cell imbalances.

Over 21–360 days after acute COVID-19, previously hospitalized patients, especially those with severe acute disease, exhibited persistent signals of systemic inflammation and tissue remodeling. Inflammation indices calculated from routine complete blood counts (derived from neutrophil, lymphocyte, monocyte, and platelet values) and red cell distribution width were consistently higher in severe cases, and circulating levels of active MMP-2 and MMP-9 remained elevated at late follow-up, aligning with a pro-inflammatory cytokine profile.

These convergent markers support an affordable, scalable framework for post-COVID care: first-line triage and longitudinal monitoring using complete blood count- based indices plus high-sensitivity C-reactive protein, followed by targeted measurement of MMP-2 and MMP-9 to refine risk of vascular and mesenchymal remodeling. The same measures offer objective inclusion and response criteria for therapeutic studies aimed at reducing inflammatory tone or modulating matrix metalloproteinase activity. Although external validation is warranted, the internal consistency across hematology, cytokines, and matrix-remodeling enzymes suggests that this panel is a clinically relevant tool for characterizing post-acute biological alterations after COVID-19 and for informing the design of future interventional studies.

Beyond our longitudinal analyses using GEE models, future studies should also explore survival methods to model the time-dependent dynamics of symptom resolution and persistence in relation to these biomarkers. In particular, hazard-based approaches, such as time-to-event models for long COVID symptoms, applied in larger multicenter cohorts with detailed time-to-event symptom data, may better characterize the prognostic value of inflammatory markers, metalloproteinases, and hematological indices for post-acute COVID-19 trajectories.

## Conclusion

Over 21–360 days after acute COVID-19, previously hospitalized patients, particularly those with severe acute disease, exhibited persistent alterations in inflammatory and matrix-remodeling biomarkers, with higher hematological inflammation indices and sustained elevations in active MMP-2 and MMP-9 compared with moderate cases and controls. These findings describe exploratory, unadjusted associations between prior severe COVID-19 and post-acute biomarker patterns and should not be interpreted as demonstrating independent biomarker effects, mechanisms specific to clinically defined long COVID syndromes, or tools for risk stratification or biomarker-guided care. Instead, the reported biomarker trajectories provide a biologic signature that may inform future hypothesis-driven research on post-acute COVID-19 sequelae. Larger, multicenter studies with harmonized follow-up timing, adjustment for key baseline covariates, and systematic, symptom-based clinical endpoints are required before these biomarkers can be considered clinically useful or predictive of long-term outcomes.

## Data Availability

The original contributions presented in the study are included in the article/Supplementary material. Further inquiries can be directed to the corresponding author.

## Glossary

ACE2: angiotensin-converting enzyme 2
AISI: Aggregate Index of Systemic Inflammation
ALP: Alkaline Phosphatase
ALT: Alanine Aminotransferase
ARDS: acute respiratory distress syndrome
AST: Aspartate Aminotransferase
BANDS: Band neutrophils
BAS: Basophils
BMI: body mass index
CLR: C-Reactive Protein to Lymphocyte Ratio
COLT: Cholesterol
COPD: chronic obstructive pulmonary disease
CPK: Creatine Phosphokinase
CREA: Creatinine
CRP: C-reactive protein
CSF: Cerebrospinal fluid
COVID-19: Coronavirus disease
ECM: extracellular matrix
EDTA: Ethylenediaminetetraacetic Acid
FT: Ferritin
GEE: Generalized Estimating Equations
GGT: Gamma-Glutamyl Transferase
HB: Hemoglobin
HCT: Hematocrit
Hcy: homocysteine
HDL: High-Density Lipoprotein
ICU: intensive care unit
IFN: Interferon
IL: Interleukin
LCR: Lymphocyte to C-Reactive Protein Ratio
LDL: Low-Density Lipoprotein
LMR: Lymphocyte to Monocyte Ratio
LSD: least significant difference
LYMP: Lymphocytes
MCH: Mean Corpuscular Hemoglobin
MCHC: Mean Corpuscular Hemoglobin Concentration
MCV: Mean Corpuscular Volume
MLR: Monocyte to Lymphocyte Ratio
MMP: matrix metalloproteinase
MNR: Monocyte to Neutrophil Ratio
MON: Monocytes
NEU: Neutrophils
NLR: Neutrophil to Lymphocyte Ratio
NLPR: Neutrophil to Lymphocyte and Platelet Ratio
NPR: Neutrophil to Platelet Ratio
N_HDL: Non-High-Density Lipoprotein
PE: Phycoerythrin
PLR: Platelet to Lymphocyte Ratio
PLT: Platelet Count
RBC: Red Blood Cell Count
RDW: Red Cell Distribution Width
RPR: Reticulocyte to Platelet Ratio
RT-PCR: Reverse Transcription-Polymerase Chain Reaction
SARS-CoV-2: Severe Acute Respiratory Syndrome Coronavirus 2
SDS: Sodium Dodecyl Sulfate
SII: Systemic Inflammation Index
SIRI: Systemic Inflammatory Response Index
SPO_2_: Saturation of Peripheral Oxygen
TG: Triglycerides
TNF: Tumor Necrosis Factor
WBC: White Blood Cell Count
