# Supplementary material for: Longitudinal trajectories of hematological indices and serum metalloproteinases-2 and 9 over 1 year after moderate and severe COVID-19
Source: Front Med (Lausanne). 2026 Jun 12;13:1824883. doi: 10.3389/fmed.2026.1824883 (PMC13303364; doi:10.3389/fmed.2026.1824883)
Supplement: Supplementary file 1 [file table_1.docx]

**Supplementary table 1.** **Hematological indices in different disease severities and timepoints of sample collection.**

| **Ratios** |  | **A_21-30_** | **A_31-90_** | **A_91-180_** | **A_181-360_** | **General (All assessments)** | **GEE (p values)** | | |  |
| --- | --- | --- | --- | --- | --- | --- | --- | --- | --- | --- |
|  | **Groups** | **Mean (CI 95%)** | **Mean (CI 95%)** | **Mean (CI 95%)** | **Mean (CI 95%)** | **Mean (CI 95%)** | **Group** | **Assessment** | **Group * Assessment** |  |
| AISI | Moderate COVID (n=22) | 283362.33  (174645.65 – 459755.01) | 223049.23  (181754.95 – 273725.46) | 305935.55  (211071.60 – 443435.13)^*^ | 214210.80  (165743.78 – 276850.60) | 253690.11  (204478.63 – 314745.23) | <0.001 | 0.003 | 0.011 |  |
|  | Severe COVID (n=18) | 369048.37  (281110.60 – 484495.08)^*^ | 275269.83  (203392.88 – 372547.35)^*a^ | 385103.04  (270835.04 – 547581.86)^*b^ | 281793.14  (209254.43 – 379477.61)^*c^ | 324031.57  (249089.14 – 421521.60) |  |  |  |  |
|  | Control (n=30) | 173186.84  (143211.93 – 209435.64) | 173186.84  (143211.93 – 209435.64) | 173186.84  (143211.93 – 209435.64) | 173186.84  (143211.93 – 209435.64) | 173186.84  (143211.93 – 209435.64) |  |  |  |  |
|  | Total Sample (n=70) | 262611.32  (215950.61 – 319354.05) | 219899.75  (191692.75 – 252257.34) | 273258.66  (227811.15 – 327772.78) | 218656.41  (189050.63 – 252898.53) |  |  |  |  |  |
|  | | | | | | | | | |  |
| CLR | Moderate COVID (n=22)  Severe COVID (n=18)  Control (n=30)  Total Sample (n=70) | 0.00179 (0.00089 – 0.00359)  0.00339 (0.00215 – 0.00534)^*^  0.00056 (0.00034 – 0.00092)  0.00151 (0.00109 – 0.00208) | 0.00096 (0.00059 – 0.00153)^*^  0.00271 (0.00173 – 0.00425)^*^  0.00056 (0.00034 – 0.00092)  0.00114 (0.00087 – 0.00149) | 0.00103 (0.00061 – 0.00176)  0.00867 (0.00235 – 0.03192)  0.00056 (0.00034 – 0.00092)  0.00172 (0.00105 – 0.00283) | 0.00638 (0.00148 – 0.02745)  0.00233 (0.00158 – 0.00342)^*^  0.00056 (0.00034 – 0.00092)  0.00203 (0.00120 – 0.00345) | 0.00183 (0.00112 – 0.00301)  0.00369 (0.00221 – 0.00616)  0.00056 (0.00034 – 0.00092) | <0.001 | 0.051 | 0.008 |  |
| LCR | Moderate COVID (n=22) | 5.78 (1.78 – 9.77) | 5.23 (2.14 – 8.32) | 5.06 (2.51 – 7.61) | 3.95 (2.03 – 5.88) | 5.01 (2.60 – 7.41) | 0.018 | 0.269 | 0.282 |  |
|  | Severe COVID (n=18) | 2.02 (0.24 – 3.81) | 1.48 (0.26 – 2.71) | 3.23 (0.18 – 6.29) | 1.27 (0.48 – 2.07) | 2.00 (0.45 – 3.55) |  |  |  |  |
|  | Control (n=30) | 9.13 (2.74 – 15.51) | 9.13 (2.74 – 15.58) | 9.13 (2.74 – 15.51) | 9.13 (2.74 – 15.51) | 9.13 (2.74 – 15.51) |  |  |  |  |
|  | Total Sample (n=70) | 5.64 (3.06 – 8.22) | 5.28 (2.88 – 7.68) | 5.81 (3.30 – 8.32) | 4.78 (2.54 – 7.02) |  |  |  |  |  |
|  | | | | | | | | | |  |
| LMR | Moderate COVID (n=) | 4.18 (3.56 – 4.91) | 3.94 (3.48 – 4.47) | 3.71 (3.17 – 4.35) | 4.27 (3.66 – 4.98) | 4.02 (3.58 – 4.52)^*^ | 0.035 | 0.275 | 0.376 |  |
|  | Severe COVID (n=18) | 3.66 (3.17 – 4.22) | 4.14 (3.62 – 4.73) | 3.98 (3.42 – 4.65) | 4.15 (3.70 – 4.66) | 3.98 (3.57 – 4.43)^*^ |  |  |  |  |
|  | Control (n=30) | 4.76 (4.28 – 5.30) | 4.76 (4.28 – 5.30) | 4.76 (4.28 – 5.30) | 4.76 (4.28 – 5.30) | 4.76 (4.28 – 5.30) |  |  |  |  |
|  | Total Sample (n=233) | 4.18 (3.85 – 4.53) | 4.27 (3.98 – 4.58) | 4.13 (3.81 – 4.48) | 4.39 (4.08 – 4.72) |  |  |  |  |  |
|  | | | | | | | | | |  |
| MLR | Moderate COVID (n=22) | 0.30 (0.20 – 0.47) | 0.28 (0.23 – 0.34) | 0.32 (0.25 – 0.40) | 0.28 (0.21 – 0.39) | 0.30 (0.23 – 0.38) | 0.034 | 0.162 | 0.350 |  |
|  | Severe COVID (n=18) | 0.30 (0.25 – 0.35) | 0.26 (0.22 – 0.30) | 0.27 (0.23 – 0.32) | 0.25 (0.22 – 0.29) | 0.27 (0.24 – 0.30) ^*^ |  |  |  |  |
|  | Control (n=30) | 0.22 (0.20 – 0.25) | 0.22 (0.20 – 0.25) | 0.22 (0.20 – 0.25) | 0.22 (0.20 – 0.25) | 0.22 (0.20 – 0.25) |  |  |  |  |
|  | Total Sample (n=70) | 0.27 (0.23 – 0.32) | 0.25 (0.23 – 0.27) | 0.27 (0.24 – 0.30) | 0.25 (0.22 – 0.28) |  |  |  |  |  |
|  | | | | | | | | | |  |
| MNR | Moderate COVID (n=22) | 0.19 (0.16 – 0.23) | 0.20 (0.15 – 0.26) | 0.20 (0.15 – 0.25) | 0.19 (0.15 – 0.25) | 0.19 (0.15 – 0.24) | 0.059 | 0.974 | 0.972 |  |
|  | Severe COVID (n=18) | 0.15 (0.13 – 0.18) | 0.15 (0.12 – 0.18) | 0.15 (0.13 – 0.18) | 0.15 (0.12 – 0.18) | 0.15 (0.13 – 0.17) |  |  |  |  |
|  | Control (n=30) | 0.14 (0.13 – 0.16) | 0.14 (0.13 – 0.16) | 0.14 (0.13 – 0.16) | 0.14 (0.13 – 0.16) | 0.14 (0.13 – 0.16) |  |  |  |  |
|  | Total Sample (n=70) | 0.16 (0.15 – 0.17) | 0.16 (0.14 – 0.18) | 0.16 (0.14 – 0.18) | 0.16 (0.14 – 0.18) |  |  |  |  |  |
|  | | | | | | | | | |  |
| NLPR | Moderate COVID (n=22) | 0.62 (0.46 – 0.78) | 0.60 (0.48 – 0.71) | 0.68 (0.57 – 0.80) | 1.97 (-0.68 – 4.64) | 0.97 (0.31 – 1.62) | 0.527 | 0.422 | 0.754 |  |
|  | Severe COVID (n=18) | 0.80 (0.63 – 0.97) | 0.75 (0.55 – 0.95) | 0.82 (0.59 – 1.05) | 0.75 (0.58 – 0.92) | 0.78 (0.62 – 0.94) |  |  |  |  |
|  | Control (n=30) | 0.70 (0.59 – 0.80) | 0.70 (0.59 – 0.80) | 0.70 (0.59 – 0.80) | 0.70 (0.59 – 0.80) | 0.70 (0.59 – 0.80) |  |  |  |  |
|  | Total Sample (n=70) | 0.71 (0.62 – 0.79) | 0.68 (0.60 – 0.77) | 0.73 (0.64 – 0.82) | 1.14 (0.25 – 0.03) |  |  |  |  |  |
|  | | | | | | | | | |  |
| NLR | Moderate COVID (n=22) | 1.54 (1.23 – 1.91) | 1.52 (1.26 – 1.83) | 1.85 (1.45 – 2.34) | 1.40 (1.18 – 1.66) | 1.57 (1.38 – 1.77) | 0.066 | 0.045 | 0.166 |  |
|  | Severe COVID (n=18) | 2.08 (1.75 – 2.47) | 1.80 (1.54 – 2.09) | 1.94 (1.64 – 2.30) | 1.78 (1.56 – 2.03) | 1.90 (1.68 – 2.14) |  |  |  |  |
|  | Control (n=30) | 1.62 (1.42 – 1.85) | 1.62 (1.42 – 1.85) | 1.62 (1.42 – 1.85) | 1.62 (1.42 – 1.85) | 1.62 (1.42 – 1.85) |  |  |  |  |
|  | Total Sample (n=70) | 1.73 (1.56 – 1.92) | 1.64 (1.50 – 1.80) | 1.80 (1.61 – 2.00) | 1.59 (1.46 – 1.73)^a,c^ |  |  |  |  |  |
|  |  |  |  |  |  |  |  |  |  |  |
|  | Moderate COVID (n=22) | 13.06 (11.28 – 15.11) | 11.07 (10.05 – 12.20) | 12.45 (10.40 – 14.89) | 12.31 (10.56 – 14.36) | 12.20 (11.09 – 13.41) |  |  |  |  |
| NPR | Severe COVID (n=18) | 16.73 (14.20 – 19.71) | 15.89 (13.08 – 19.32) | 16.89 (14.36 – 19.87) | 16.28 (13.61 – 19.47) | 16.44 (14.32 – 18.88)^#^ | 0.002 | 0.355 | 0.204 |  |
|  | Control (n=30) | 13.58 (11.72 – 15.74) | 13.58 (11.72 – 15.74) | 13.58 (11.72 – 15.74) | 13.58 (11.72 – 15.74) | 13.58 (11.72 – 15.74) |  |  |  |  |
|  | Total Sample (n=70) | 14.37 (13.16 – 15.69) | 13.37 (12.25 – 14.60) | 14.19 (12.91 – 15.59) | 13.96 (12.73 – 15.32) |  |  |  |  |  |
|  | Moderate COVID (n=22) | 0.11 (0.10 – 0.13) | 0.13 (0.11 – 0.16) | 0.15 (0.11 – 0.20)^a^ | 0.59 (0.13 – 2.66) | 0.19 (0.13 – 0.28) |  |  |  |  |
| PLR | Severe COVID (n=18) | 0.13 (0.10 – 0.15) | 0.12 (0.10 – 0.14) | 0.12 (0.10 – 0.14) | 0.11 (0.10 – 0.13) | 0.12 (0.10 – 0.14) | 0.083 | 0.115 | 0.041 |  |
|  | Control (n=30) | 0.12 (0.11 – 0.14) | 0.12 (0.11 – 0.14) | 0.12 (0.11 – 0.14) | 0.12 (0.11 – 0.14) | 0.12 (0.11 – 0.14) |  |  |  |  |
|  | Total Sample (n=70) | 0.12 (0.11 – 0.13) | 0.12 (0.11 – 0.14) | 0.13 (0.12 – 0.15) | 0.20 (0.12 – 0.34) |  |  |  |  |  |
| RPR | \| Moderate COVID (n=22) \| 0.052 (0.045 – 0.059) \| 0.049 (0.045 – 0.055) \| 0.052 (0.046 – 0.058) \| 0.052 (0.046 – 0.060) \| 0.051 (0.046 – 0.057) \|  \|  \|  \| \| --- \| --- \| --- \| --- \| --- \| --- \| --- \| --- \| --- \| \| Severe COVID (n=18) \| 0.059 (0.050 – 0.069) \| 0.061 (0.048 – 0.079) \| 0.062 (0.046 – 0.084) \| 0.060 (0.049 – 0.072) \| 0.061 (0.050 – 0.074) \| 0.324 \| 0.869 \| 0.786 \| \| Control (n=30) \| 0.055 (0.051 – 0.061) \| 0.055 (0.051 – 0.061) \| 0.055 (0.051 – 0.061) \| 0.055 (0.051 – 0.061) \| 0.055 (0.051 – 0.061) \|  \|  \|  \| \| Total Sample (n=70) \| 0.055 (0.051 – 0.060) \| 0.055 (0.050 – 0.061) \| 0.056 (0.050 – 0.063) \| 0.056 (0.051 – 0.061) \|  \|  \|  \|  \| | 0.052 (0.045 – 0.059)  0.059 (0.050 – 0.069)  0.055 (0.051 – 0.061)  0.055 (0.051 – 0.060) | 0.049 (0.045 – 0.055)  0.061 (0.048 – 0.079)  0.055 (0.051 – 0.061)  0.055 (0.050 – 0.061) | 0.052 (0.046 – 0.058)  0.062 (0.046 – 0.084)  0.055 (0.051 – 0.061)  0.056 (0.050 – 0.063) | 0.052 (0.046 – 0.060)  0.060 (0.049 – 0.072)  0.055 (0.051 – 0.061)  0.056 (0.051 – 0.061) | 0.051 (0.046 – 0.057)  0.061 (0.050 – 0.074)  0.055 (0.051 – 0.061) | 0.324 | 0.869 | 0.786 |  |
| SII | Moderate COVID (n=22)  Severe COVID (n=18)  Control (n=30)  Total Sample (n=70) | 401.14 (315.12 – 510.63)  577.86 (451.85 – 739.01)^#*^  390.48 (322.91 – 458.01)  448.99 (395.61 – 509.58) | 413.56 (336.66 – 508.03)  474.76 (379.92 – 593.29)^a^  390.48 (322.91 – 458.01)  424.82 (378.96 – 476.23) | 532.48 (390.49 – 726.10)  565.37 (426.28 – 749.85)^*^  390.48 (322.91 – 458.01)  489.87 (421.81 – 568.91)^b^ | 364.31 (299.60 – 442.99)^c^  479.43 (380.60 – 603.91)  390.48 (322.91 – 458.01)  408.57 (364.55 – 457.91)^c^ | 423.55 (361.50 – 496.25)  522.20 (421.16 – 647.48)  390.48 (322.91 – 458.01) | 0.324 | 0.869 | 0.786 |  |
| SIRI | Moderate COVID (n=22)  Severe COVID (n=18)  Control (n=30)  Total Sample (n=70) | 1134.62 (631.44 – 2038.78)  1327.77 (1051.40 – 1676.79)^*^  722.08 (606.64 – 859.49) | 827.27 (682.95 – 1002.10)  1019.78 (797.83 – 1303.49)^*a^  722.08 (606.64 – 859.49) | 1063.58 (793.59 – 1425.42)^*^  1285.17 (966.26 – 1709.31)^*^  722.08 (606.64 – 859.49) | 896.20 (601.37 – 1335.56)  1025.35 (831.07 – 1265.03)^*a,c^  722.08 (606.64 – 859.49) | 972.57 (735.80 – 1285.52)  1155.75 (938.84 – 1422.78)  722.08 (606.64 – 859.49) | 0.002 | 0.001 | 0.008 |  |
|  | |  |  |  |  |  |  |  |  |  |

Abbreviations: A_21-30_: assessment conducted between 21 and 30 days after symptom onset or hospital discharge for severe COVID; A_31-90_: assessment conducted between 31 and 90 days after symptom onset or hospital discharge for severe COVID; A_91-180_: assessment conducted between 91 and 180 days after symptom onset or hospital discharge for severe COVID; and A_181-360_: assessment conducted between 181 and 360 days after symptom onset or hospital discharge for severe COVID. The control group was assessed only once. **AISI:** Aggregate Index of Systemic Inflammation; **CPR_Ly:** C-Reactive Protein to Lymphocyte ratio; **LCR:** Lymphocyte to C-Reactive Protein Ratio; **LMR:** Lymphocyte to Monocyte Ratio; **MLR:** Monocyte to Lymphocyte Ratio; **MNR:** Monocyte to Neutrophil Ratio; **NLPR:** Neutrophil to Lymphocyte and Platelet Ratio; **NLR:** Neutrophil to Lymphocyte Ratio; **NPR:** Neutrophil to Platelet Ratio; **PLR:** Platelet to Lymphocyte Ratio; **RPR:** Red cell distribution width-to-platelet ratio; **SII:** Systemic Immune-inflammation Index; **SIRI:** Systemic Inflammation Response Index. GEE, Generalized estimating equation. * Different from the control group in the respective assessment (p <0.05); # Different from the moderate COVID in the respective assessment (p <0.05); a Different from assessment 1 (p <0.05); b Different from assessment 2 (p <0.05); c Different from assessment 3 (p <0.05).

| **Supplementary table 2.** **Gelatinase levels activity in different disease severity and timepoints of sample collection.** | | | | | | | | | | | |
| --- | --- | --- | --- | --- | --- | --- | --- | --- | --- | --- | --- |
| **MMPs** |  | **A_21-30_** | **A_31-90_** | | | **A_91-180_** | **A_181-360_** | **General (All assessments)** | **GEE (p values)** | | |
|  | **Groups** | **Mean (CI 95%)** | **Mean (CI 95%)** | | | **Mean (CI 95%)** | **Mean (CI 95%)** | **Mean (CI 95%)** | **Group** | **Assessment** | **Group * Assessment** |
| PRO  MMP-2 | Moderate COVID (n=22) | 500517.00  (457617.66 – 547437.94) | | | 449730.77 (417695.94 – 484222.49) | 443850.56  (398397.05 – 494489.92) | 453996.03  (413370.31 – 498614.41) | 461492,94 (438730,86 – 485435,96) | <0.001 | 0.054 | 0.083 |
|  | Severe COVID (n=18) | 644106.75 (586010.26 – 707962.87) | | | 590492,31  (549712.42 – 634297.42) | 629408.07  (583373.47 – 679075.30) | 678335.06  (619321.28 – 742972.14) | 634800.36  (609183.67 – 661494.26)^#*^ |  |  |  |
|  | Control (n=30) | 482145.3  (447539.42 – 519426.94) | | | 482145.3  (447539.42 – 519426.94) | 482145.3  (447539.42 – 519426.94) | 482145.3  (447539.42 – 519426.94) | 482145.23 (447539.42 – 519426.93) |  |  |  |
|  | Total Sample (n=70) | 537672.95 (511443.35–565247.74) | | | 504020.55  (483129.04 – 525815.45) | 512604.39  (487341.20 – 539177.19) | 529531.01  (503638.65 – 556754.51) |  |  |  |  |
|  | | | | | | | | | | | |
| ACTIVE  MMP-2 | Moderate COVID (n=22) | 922116,94  (800040,02 – 1062821.39)^*^ | | | 800040.02  (1018246.51 –1179166.30)^a*^ | 1041099.69  (963611.04 – 1124819.57)^a*^ | 1301397.18  (1170584.53 – 1446828.14)^a^ | 1081684.34  (1020002.34 – 1147096.40) | <0.001 | <0.001 | <0.001 |
|  | Severe COVID (n=18) | 1226897.85  (1163684.65 – 1293544.89)^*^ | | | 1466354.69  (1314777.04 – 1635407.38)^*^ | 1291603.27  (1203854.33 – 1385748.22)^*^ | 1490619.28  (1335961.26 – 1663181.34)^c^ | 1364224.44  (1304855.14 – 1426294.97) |  |  |  |
|  | Control (n=30) | 1319884.28  (1230990.80 – 1415197.01) | | | 1319884.28  (1230990.80 – 1415197.01) | 1319884.28  (1230990.80 – 1415197.01) | 1319884.28  (1230990.80 – 1415197.01) | 1319884.28  (1230990.80 – 1415197.01) |  |  |  |
|  | Total Sample (n=70) | 1142992.60  (1081171.91 – 1208348.16) | | | 1284782.54  (1222601.34 – 1350126.26) | 1210744.31  (1161072.60 – 1262541.01) | 1368056.87  (1293732.56 – 1446651.06) |  |  |  |  |
|  |  |  |  |  |  |  |  |  |  |  |  |
| PRO  MMP-9 | Moderate COVID (n=22) | 592834.21  (540220.11 – 650572.60)^*^ | | | 473615.74  (419438.58 – 534790.75)^a^ | 639272.84  (556153.38 – 734814.84)^b*^ | 491229.82  (435708.74 – 553825.78)^a,c^ | 544919.80  (510405.37 – 581768.15) | <0.001 | 0.002 | <0.001 |
|  | Severe COVID (n=18) | 665965.45  (581859.02 – 762229.28)^*^ | | | 633432,34  (584591.31 – 686353.91)^#*^ | 679265.67  (621141.25 – 742829.18)^*^ | 866500.87  (795081.97 –944335.04)^a,b,c#*^ | 705895.07  (667781.91 – 746183.52) |  |  |  |
|  | Control (n=30) | 518255.24  (477245.17 – 562789.34) | | | 518255.24  (477245.17 – 562789.34) | 518255,24  (477245.17 – 562789.34) | 518255.24  (477245.17 – 562789.34) | 518255.24  (477245.17 – 562789.34) |  |  |  |
|  | Total Sample (n=70) | 589263.52  (554306.07 – 626425.57) | | | 537720.62  (508553.35 – 568560.73) | 608260.86  (571898.04 – 646935.73) | 604225.63  (571117.78 – 639252.75) |  |  |  |  |
|  | | | | | | | | | | | |
| ACTIVE  MMP-9 | Moderate COVID (n=22) | 592834.21  (540220.11 – 650572.60) | | 473615.74  (419438.58 – 534790.75)^a^ | | 639272.84  (556153.38 – 734814.84)^a*^ | 491229.82  (435708.74 – 553825.78)^a^ | 544919.80  (510405.37 – 581768.15) | <0.001 | <0.001 | <0.001 |
|  | Severe COVID (n=18) | 665965.45  (581859.02–762229.28)^#*^ | | 633432.34  (584591.31 – 686353.91)^#*^ | | 679265.67  (621141.25 – 742829.18)^a,b#^ | 866500.87  (795081.97 – 944335.04)^c#^ | 705895.07  (667781.91 – 746183.52) |  |  |  |
|  | Control (n=30) | 518255.24  (477245.17 – 562789.34) | | 518255,24  (477245.17 – 562789.34) | | 518255,24  (477245.17 – 562789.34) | 518255,24  (477245.17 – 562789.34) | 518255.24  (477245.17 – 562789.34) |  |  |  |
|  | Total Sample (n=70) | 589263.52  (554306.07 – 626425.57) | | 537720.62  (508553.35 – 568560.73) | | 608260.86  (571898.04 – 646935.73) | 604225.63  (571117.78 – 639252.75) |  |  |  |  |
|  | | | | | | | | | | | |
|  | | | | | | | | | | | |

Abbreviations: A_21-30_: assessment conducted between 21 and 30 days after symptom onset or hospital discharge for severe COVID; A_31-90_: assessment conducted between 31 and 90 days after symptom onset or hospital discharge for severe COVID; A_91-180_: assessment conducted between 91 and 180 days after symptom onset or hospital discharge for severe COVID; and A_181-360_: assessment conducted between 181 and 360 days after symptom onset or hospital discharge for severe COVID. The control group was assessed only once. **Pro MMP-2**: Pro-Matrix Metalloproteinase-2; **Active MMP-2**: Active Matrix Metalloproteinase-2; **Pro MMP-9**: Pro-Matrix Metalloproteinase-9; **Active MMP-9**: Active Metalloproteinase-9. GEE, Generalized estimating equation. * Different from the control group in the respective assessment (p <0.05); # Different from the moderate COVID in the respective assessment (p <0.05); a Different from assessment 1 (p <0.05); b Different from assessment 2 (p <0.05); c Different from assessment 3 (p <0.05).
