# Supplementary material for: Longitudinal trajectories of hematological indices and serum metalloproteinases-2 and 9 over 1 year after moderate and severe COVID-19
Source: Front Med (Lausanne). 2026 Jun 12;13:1824883. doi: 10.3389/fmed.2026.1824883 (PMC13303364; doi:10.3389/fmed.2026.1824883)
Supplement: Supplementary file 2 [file table_2.docx]

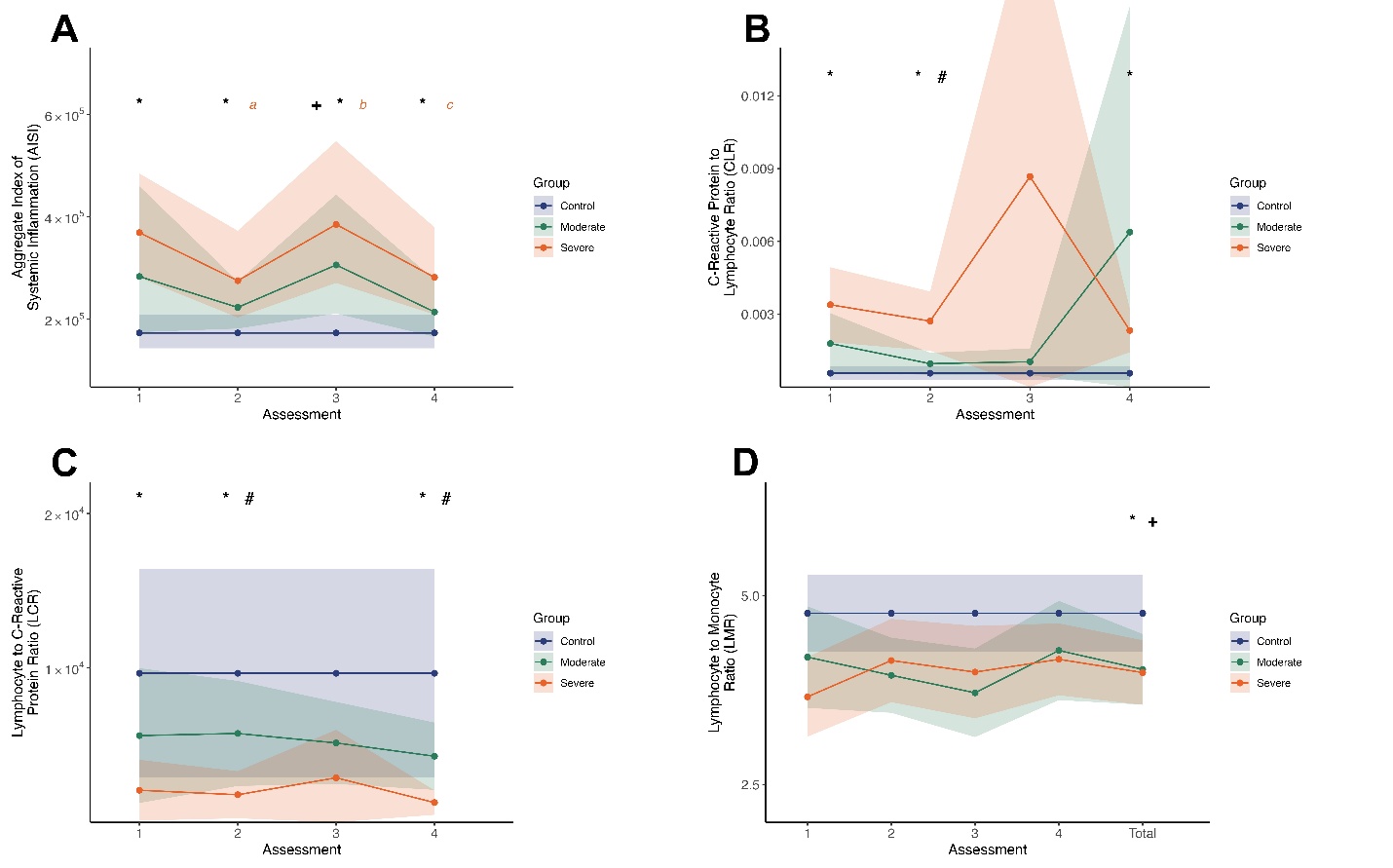


**Figure 2a.** Hematological index trajectories expressed as mean values (±95% CI) across assessment time points according to disease severity groups (Control, Moderate COVID-19, and Severe COVID-19) for the evaluated outcomes (A–D), demonstrating between-group differences and longitudinal changes over time. Comparison of (A) Aggregate Index of Systemic Inflammation (AISI), (B) C-reactive protein-to-lymphocyte ratio (CLR), (C) Lymphocyte-to-C-reactive protein ratio (LCR), (D) Lymphocyte-to-monocyte ratio (LMR). * indicates difference between control and severe groups. # indicates difference between moderate and severe groups. + indicates difference between control and moderate groups. a indicates difference to assessment 1, within group. b indicates difference to assessment 2, within group. c indicates difference to assessment 3, within group. Groups are represented as follows: Control (blue), Moderate COVID-19 (green), and Severe COVID-19 (orange). Graphs were generated using RStudio (RStudio PBC, Boston, MA, USA).


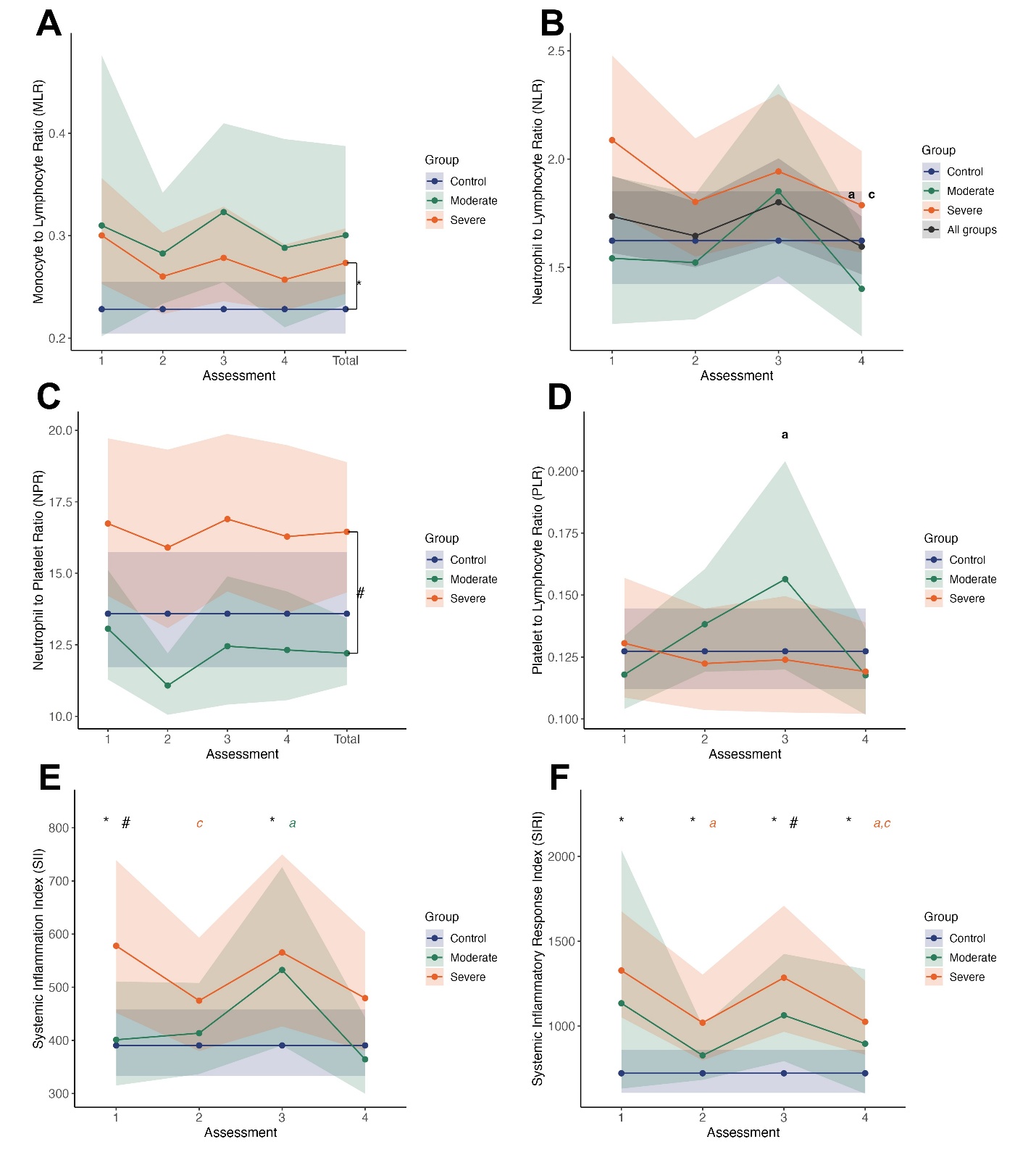
**Figure 2b. Hematological index trajectories according to disease severity and assessment time points.** Comparison of (A) Monocyte-to-Lymphocyte Ratio (MLR), (B) Neutrophil-to-Lymphocyte Ratio (NLR), (C) Neutrophil-to-Platelet Ratio (NPR), (D) Platelet-to-Lymphocyte Ratio (PLR), (E) Systemic Immune-Inflammation Index (SII), and (F) Systemic Inflammation Response Index (SIRI) among Control, Moderate COVID-19, and Severe COVID-19 groups across the four assessment periods. Data are presented as mean values ± 95% confidence interval (CI). Lines represent group trajectories over time, and shaded areas indicate the 95% CI. Groups are represented as follows: Control (blue), Moderate COVID-19 (green), Severe COVID-19 (orange), and pooled data from all groups (gray, panel B only). ). * indicates difference between control and severe groups. # indicates difference between moderate and severe groups. + indicates difference between control and moderate groups. a indicates difference to assessment 1, within group. b indicates difference to assessment 2, within group. c indicates difference to assessment 3, within group. Groups are represented as follows: Control (blue), Moderate COVID-19 (green), and Severe COVID-19 (orange). Graphs were generated using RStudio (RStudio PBC, Boston, MA, USA).

**
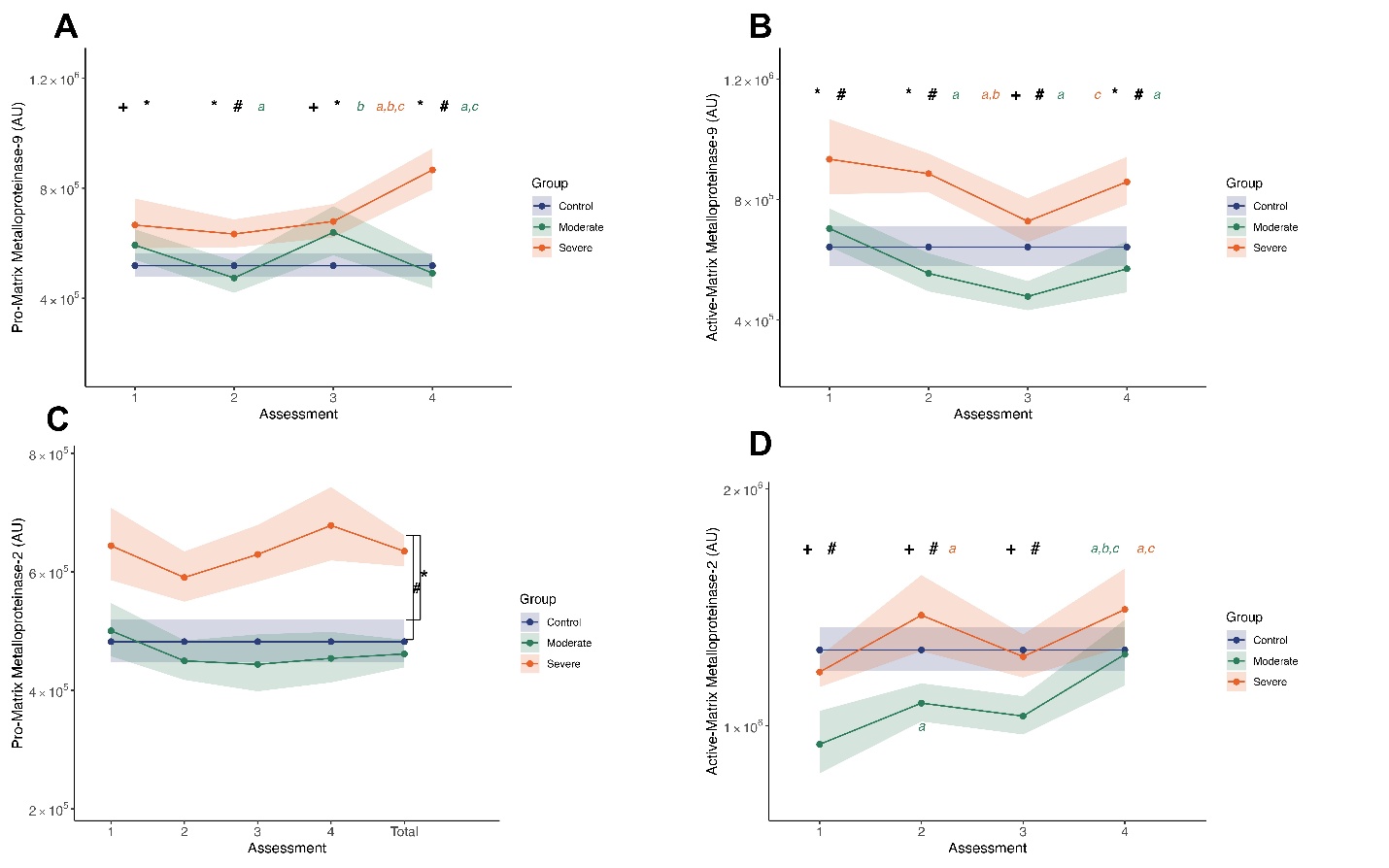
Figure 3.** Trajectories of mean scores (±95% CI) across assessments by group (control, individual, group sessions, and mixed sessions) for the four evaluated outcomes (A–D), showing between-group differences and changes over time. * indicates difference between control and severe groups. # indicates difference between moderate and severe groups. + indicates difference between control and moderate groups. a indicates difference to assessment 1, within group. b indicates difference to assessment 2, within group. c indicates difference to assessment 3, within group. Groups are represented as follows: Control (blue), Moderate COVID-19 (green), and Severe COVID-19 (orange). Graphs were generated using RStudio (RStudio PBC, Boston, MA, USA).
